# Early Endothelial Dysfunction in Type 1 Diabetes Is Accompanied by an Impairment of Vascular Smooth Muscle Function: A Meta-Analysis

**DOI:** 10.3389/fendo.2020.00203

**Published:** 2020-04-17

**Authors:** Elodie Lespagnol, Luc Dauchet, Mehdi Pawlak-Chaouch, Costantino Balestra, Serge Berthoin, Martin Feelisch, Matthieu Roustit, Julien Boissière, Pierre Fontaine, Elsa Heyman

**Affiliations:** ^1^Univ. Lille, Univ. Artois, Univ. Littoral Côte d'Opale, ULR 7369 - URePSSS - Unité de Recherche Pluridisciplinaire Sport Santé Société, Lille, France; ^2^Univ. Lille, Inserm, CHU Lille, Institut Pasteur de Lille, U1167 - RID-AGE - Facteurs de risque et déterminants moléculaires des maladies liées au vieillissement, Lille, France; ^3^Environmental and Occupational (Integrative) Physiology Laboratory, Haute École Bruxelles-Brabant HE2B, Brussels, Belgium; ^4^Clinical and Experimental Sciences, Faculty of Medicine, University Hospital Southampton NHS Foundation Trust, University of Southampton, Southampton, United Kingdom; ^5^Univ. Grenoble Alpes, HP2, Inserm, CHU Grenoble Alpes, Grenoble, France; ^6^Département d'endocrinologie, Diabète et maladies métaboliques, Hôpital Huriez, Université de Lille, Lille, France

**Keywords:** endothelial function, exercise, macrocirculation, microcirculation, peripheral vascular disease, smooth muscle function, type 1 diabetes

## Abstract

**Background:** A large yet heterogeneous body of literature exists suggesting that endothelial dysfunction appears early in type 1 diabetes, due to hyperglycemia-induced oxidative stress. The latter may also affect vascular smooth muscles (VSM) function, a layer albeit less frequently considered in that pathology. This meta-analysis aims at evaluating the extent, and the contributing risk factors, of early endothelial dysfunction, and of the possible concomitant VSM dysfunction, in type 1 diabetes.

**Methods:** PubMed, Web of Sciences, Cochrane Library databases were screened from their respective inceptions until October 2019. We included studies comparing vasodilatory capacity depending or not on endothelium (i.e., endothelial function or VSM function, respectively) in patients with uncomplicated type 1 diabetes and healthy controls.

**Results:** Fifty-eight articles studying endothelium-dependent function, among which 21 studies also assessed VSM, were included. Global analyses revealed an impairment of standardized mean difference (SMD) (Cohen's d) of endothelial function: −0.61 (95% CI: −0.79, −0.44) but also of VSM SMD: −0.32 (95% CI: −0.57, −0.07). The type of stimuli used (i.e., exercise, occlusion-reperfusion, pharmacological substances, heat) did not influence the impairment of the vasodilatory capacity. Endothelial dysfunction appeared more pronounced within macrovascular than microvascular beds. The latter was particularly altered in cases of poor glycemic control [HbA_1c_ > 67 mmol/mol (8.3%)].

**Conclusions:** This meta-analysis not only corroborates the presence of an early impairment of endothelial function, even in response to physiological stimuli like exercise, but also highlights a VSM dysfunction in children and adults with type 1 diabetes. Endothelial dysfunction seems to be more pronounced in large than small vessels, fostering the debate on their relative temporal appearance.

## Introduction

Despite significant advances in diabetes care, individuals with type 1 diabetes remain to be prone to the development of comorbidities, in particular those associated with vascular complications. Type 1 diabetes is associated with a 2- to 10-fold higher mortality and cardiovascular disease risk (1). Structural and functional endothelial aberrations seem to occur in small and large blood vessels early during diabetes development, long before the manifestation of overt micro- or macro-vascular complications (2–4). The associated endothelial dysfunction is now accepted as a reliable predictor of cardiovascular disease (5, 6). Alarming data suggest a >35% prevalence of endothelial dysfunction in individuals within 5 years of type 1 diabetes (7). Endothelial dysfunction in diabetes may be the result of a combination of multiple stressors including hyperglycaemia and oxidative stress (1, 8). Acting in concert, these factors lead to a decrease in the bioavailability of nitric oxide (NO^.^). Vascular homeostasis depends to a significant extent on the capacity of the endothelium to produce NO. While one of the principal functions that led to the discovery of NO in the cardiovascular system is to relax vascular smooth muscle by enhancing cyclic guanosine monophosphate (cGMP) production, this is only part of the story. NO is also a potent antioxidant and a regulator of local and systemic redox status (4), and these facets may play a more significant role in metabolic disease settings than hitherto assumed.

Much has been written on the subject of early endothelial dysfunction in type 1 diabetes. However, this large body of literature remains complex, with numerous contradictory findings (Table 1). It should be emphasized that the conventional assessments of endothelial function always include the responsiveness of the vascular smooth muscle (VSM) layer in addition. To better dissect the relative contribution of these two contributors to vascular dysfunction, it is crucial to specifically assess vascular smooth muscle reactivity in parallel to the endothelial function test. However, VSM function is considered in only less than a half of the papers dealing with endothelial function in type 1 diabetes, with contradictory results (Table 1). *In vitro* evidence strongly suggests a deleterious impact of chronic hyperglycemia on VSM, by provoking a dysregulation of Ca^2+^ signaling (65) and vascular remodeling (66).

**Table 1:** Main characteristics of studies included in the current meta-analysis.

**Authors and year of publication**	***n***** (% women)**		**Age (years)**		**Type 1 diabetes' duration (years)**	**Type 1 Diabetes HbA1c (%)**	**Glycaemia before test (mmol.L) (F/PP)**	**Quality scores**				**Vascular region assessed (VAR/PEAK)**	**Endothelium-dependent function (EF) Time and place of occlusion or characteristic of exercise (*technique*)**	**Endothelium independent function (VSM)**
	**Type 1 diabetes**	**HC**	**Type 1 diabetes**	**HC**			**Type 1 diabetes**	**Complications**	**Age matching**	**Gender matching**	**BMI matching**			
Abd El Dayem et al. (9)	62^†^ (50)	30^†^ (50)	16.1 ± 2.6	16.1 ± 2.6	8.9 ± 3.11	9.5 ± 1.9	NA (F)	0	2	2	1	MACRO artery (VAR)	↘FMD 5 min on forearm (*ultrasound*)	↔ NMD
Abi-Chahin et al., (10)	30^*^ (70)	31 (68)	23.7 ± 4.31	23.4 ± 5.4	12.9 ± 6.7	NA	NA (NA)	1	2	1	1	MICRO cutaneous (VAR)	↘PORH 1 min on the fourth finger (*capillaroscopy*)	NA
Aburawi et al. (11)	15 (NA)	10 (NA)	14 ± 4.0	14.0 ± 3.0	5.0 ± 3.0	7.3 ± 2.0	NA (NA)	0	2	0	0	MACRO artery (VAR)	↘FMD 5 min on forearm (*ultrasound*)	↔ NMD
Allen et al. (12)	15^†^ (0)	15^†^ (0)	29.0 ± 6.0	26.0 ± 6.0	13.0 ± 7.0	8.2 ± 1.3	11.3 ± 4.6 (PP)	0	2	1	1	MICRO cutaneous (PEAK)	↔ During intermittent local exercise (1 contraction per 4 s at 25% maximal voluntary capacity, 3 min) + FMD (*plethysmography*)	NA
													↘FMD 5 min on upper arm (*plethysmography*)	
Aslan et al. (13)	76^*^ (50)	36^*^ (45)	30.6 ± 10.3	32.4 ± 8.5	11.7 ± 8.1	8.9 ± 1.57	7.9 ± 3.1 (NA)	1	2	2	2	MACRO artery (VAR)	↘FMD 5 min on forearm (*ultrasound*)	↔ NMD
Oral contraceptive														
Babar et al. (14)	21 (57^‡^)	15 (60^‡^)	8.3 ± 1.37	7.6 ± 1.2	4.3 ± 4.6	8.0 ± 0.9	NA (F)	0	2	0	0	MACRO artery (VAR)	↘FMD 5 min on forearm (*ultrasound*)	NA
Bayir et al. (15)	50 (46^‡^)	45 (47^‡^)	12.1 ± 2.02	11.5 ± 1.9	3.7 ± 1.9	9.2 ± 2.5	NA (F)	1	2	2	1	MACRO artery (VAR)	↔ FMD 3 min on forearm (*ultrasound*)	NA
Bellien et al. (16)	16 (54)	24^*^ (50)	NA	37.0 ± 14.7	NA	NA	NA (NA)	1	0	1	1	MACRO artery (VAR)	↘FMD 10 min on the wrist (*ultrasound*)	↔ NMD
													↘Heat (*ultrasound*)	NA
Boolell and Tooke (17)	6^†^ (17)	9^†^ (22)	34.0 ± 11.0	30.0 ± 11.0	4.5 ± 2.9	7.7 ± 1.8	7.6 ± 4.0 (PP)	1	0	0	0	MICRO cutaneous (PEAK)	↔ Capsaïcine (*laser Doppler*)	NA
													↘Substance P (*laser Doppler*)	NA
Bradley et al. (18)	199^*^ (51^‡^)	178 (53^‡^)	14.4 ± 1.6	14.4 ± 2.1	7.2 ± 3.1	8.5 ± 1.2	NA (F)	1	2	0	0	MACRO artery (VAR)	↘FMD 5 min on forearm (*ultrasound*)	NA
Bruzzi et al. (19)	39 (51)	45 (51)	11.2 ± 3.7	10.2 ± 3.1	4.0 ± 2.8	8.0 ± 0.9	13.6 ± 5.3 (F)	0	2	0	1	MACRO artery (VAR)	↔ FMD 4.5 min on forearm (*ultrasound*)	NA
Calver et al. (20)	10^†^ (0)	10^†^ (0)	26.2 ± 4.7	24.9 ± 5.1	3.2 ± 3.1	6.7 ± 1.6	NA (NA)	0	2	2	0	MICRO Cutaneous + muscle(PEAK)	↔ ACh (*plethysmography*)	↘SNP
Ceriello et al. (21)	22 (11)	20 (8)	23.5 ± 13.6	23.2 ± 13.9	NA	8.1 ± 1.9	NA (F)	1	1	0	1	MACRO artery (VAR)	↘FMD 5 min on forearm (*ultrasound*)	NA
Chiesa et al. (22)	70 (61)	30 (47)	14.6 ± 1.7	13.9 ± 2.1	8.9 ± 3.8	8.3	NA (NA)	1	2	0	0	MACRO artery (VAR)	↔ FMD 5 min on forearm (*ultrasound*)	NA
Ciftel et al. (23)	42 (NA^‡^)	40 (NA^‡^)	13.2 ± 2.6	13.1 ± 2.8	6.9 ± 1.8	9.0 ± 1.4	NA (NA)	1	2	0	2	MACRO artery (VAR)	↘FMD 3 min on forearm (*ultrasound*)	NA
DiMeglio et al. (24)	17 (52)	18 (50)	10.7 ± 3.5	20.5 ± 1.4	21.1 ± 3.5	9.4 ± 1.6	NA (F)	0	0	2	0	MICRO cutaneous (VAR)	↘ACh (*laser Doppler*)	NA
Eltayeb et al. (25)	30 (43^‡^)	30 (43^‡^)	11.1 ± 3.8	9.8 ± 3.5	3.9 ± 0.6	9.7 ± 2.2	12.8 (F)	1	2	2	2	MACRO artery (VAR)	↘FMD 4.5 min on forearm (*ultrasound*)	NA
Fayh et al. (26)	20 (0)	10 (0)	23.3 ± 5.5	23.4 ± 2.6	8.5 ± 18.8	8.3 ± 1.3	10.2 ± 3.4 (F)	1	1	0	1	MICRO muscle (PEAK)	↔ Submaximal aerobic exercise immediate end (10% below VO_2_ response at ventilatory threshold, 45 min) (*plethysmography*)	NA
Franzeck et al. (27)	8^*^ (12)	10^*^ (50)	28.5 ± 5.2	25.1 ± 1.9	12.0 ± 10.9	7.4 ± 1.3	10.3 ± 5.2 (NA)	0	2	0	0	MICRO cutaneous (VAR)	FMD (NA) 4 min (*laser Doppler*)	NA
Fujii et al. (28)	12 (18)	11 (17)	25.0 ± 5.0	24.0 ± 4.0	12.5 ± 6.0	7.3 ± 0.8	NA (NA)	0	1	1	1	MICRO cutaneous (VAR)	↔ Submaximal aerobic exercise immediate end (45% VO_2peak_, 30 min) + Heat (*laser Doppler*)	↘SNP
													↔ Submaximal aerobic exercise recovery + Heat (*laser Doppler*)	↘SNP
Glowinska-Olszemska et al. (29)	52^†^ (54)	36^†^ (56)	14.5 ± 2.4	15.1 ± 2.7	6.0 ± 3.0	8.7 ± 1.5	NA (F)	1	1	2	1	MACRO artery (VAR)	↘FMD 4 min on forearm (*ultrasound*)	NA
Gomes et al. (30)	50^†^ (42)	46^†^ (48)	32.8 ± 1.66	NA	15.0 ± 1.3	NA	NA (PP)	0	2	2	2	MICRO cutaneous (PEAK)	↘ACh (*laser Doppler*)	↘SNP
													↔ PORH (*laser Doppler*)	NA
													↔ Heat (*laser Doppler*)	NA
Grzelak et al. (31) (3 cohorts)	10 (0)	21 (0)	24.3	24.2	NA	NA	6.4 (NA)	0	1	0	0	MACRO artery (VAR)	FMD (NA) 5 min on forearm (*ultrasound*)	NA
													Intermittent local exercise immediate end (30 cycles of exercise: ~30 times within 30 s) (NA) (*ultrasound*)	
	10 (0)	21 (0)	38.6	37.7	NA	NA	7.2 (NA)	0	1	0	0	MACRO artery (VAR)	FMD (NA) 5 min on forearm (*ultrasound*)	NA
													Intermittent local exercise immediate end (30 cycles of exercise: ~30 times within 30 s) (NA) (*ultrasound*)	
	11 (0)	29 (0)	53.2	52.1	NA	NA	6.8 (NA)	0	1	0	0	MACRO artery (VAR)	FMD (NA) 5 min on forearm (*ultrasound*)	NA
													Intermittent local exercise immediate end (30 cycles of exercise: ~30 times within 30 s) (NA) (*ultrasound*)	
Haak et al. (32)	9^*^ (56)	9^*^ (45)	33.3 ± 1.0	27.4 ± 1.1	11.4 ± 3.0	7.2 ± 0.2	NA (F)	1	0	0	0	MICRO cutaneous (PEAK)	PORH (NA) 3 min on arm (*capillaroscopy*)	NA
Heier et al. (33)	46 (48)	32 (53)	2.0 ± 0.6	2.2 ± 0.6	10.0	8.7 ± 1.4	NA (NA)	1	1	0	1	MICRO cutaneous (VAR)	↔ PORH 5 min on forearm (*plethysmography*)	NA
Hoffman et al. (34)	25 (60)	29 (48)	15.1 ± 2.2	14.5 ± 2.0	5.6	7.6	NA (PP)	2	2	2	2	MICRO cutaneous (PIC)	↔ PORH 4 min on arm (*capillaroscopy*)	NA
Järvisalo et al. (2)	45^†^ (33)	30^†^ (40)	11.0 ± 2.0	11.0 ± 2.0	4.4 ± 2.9	8.9 ± 1.4	12.2 ± 4.5 (F)	1	2	2	1	MACRO artery artery (VAR)	↘FMD 4.5 min on forearm (*ultrasound*)	↔ NMD
Johnstone et al. (35)	15^*^ (73)	16^*^ (75)	30.0 ± 3.9	31.0 ± 8.0	14.0 ± 7.7	11.9 ± 2.3	NA (PP)	0	1	1	0	MICRO Cutaneous + muscle (PEAK)	↘MCh (*plethysmography*)	↔ SNP
												MICRO cutaneous (PEAK)	↔ occlusion-reperfusion 5 min on upper arm (*plethysmography*)	NA
Khan et al. (36)	55^*^ (59)	25 (52)	14.8 ± 3.7	15.4 ± 4.5	6.6 ± 4.5	8.7 ± 1.5	NA (PP)	0	1	0	1	MICRO cutaneous (VAR)	↘ACh (*laser Doppler*)	↘SNP
													↘Heat (*laser Doppler*)	NA
Koïtka et al. (37)	12^†^ (50)	12^†^ (67)	22.0 ± 3.5	23.0 ± 3.5	8.9 ± 6.2	9.2 ± 2.8	NA (PP)	0	2	0	1	MICRO cutaneous (PEAK)	↘ACh (*laser Doppler*)	↔ SNP
Lockhart et al. (38)	40^*^ (NA)	32^*^ (NA)	40.0 ± 12.0	40.4 ± 12.3	NA	8.1 ± 1.2	NA (F)	0	2	2	0	MACRO artery (VAR)	↘FMD 5 min on forearm (*ultrasound*)	NMD
Lytvyn et al. (39)	188 (51^‡^)	65 (57^‡^)	14.4 ± 1.7	14.0 ± 2.0	7.2 ± 3.2	8.5 ± 1.3	NA (NA)	0	0	0	0	MACRO artery (VAR)	↔ FMD 5 min on forearm (*ultrasound*)	NA
Lytvyn et al. (40)	49 (51)	24 (50)	26.3 ± 5.4	25.5 ± 4.5	14.3 ± 7.2	7.8 ± 1.3	NA (NA)	0	0	0	0	MACRO artery (VAR)	FMD (NA) 5 min on forearm (*ultrasound*)	NMD (NA)
Mackenzie et al. (41)	122 (43)	33 (61)	14.1 ± 2.9	14.2 ± 3.6	5.3 ± 3.6	8.7 ± 1.3	13.4 ± 4.7 (F)	0	2	2	1	MACRO artery (VAR)	↘FMD 4 min on forearm (*ultrasound*)	↘NMD
Maftei et al. (42)	167 (NA)	57 (NA)	NA	NA	NA	NA	NA (NA)	1	2	2	0	MACRO artery (VAR)	↘FMD 5 min on forearm (*ultrasound*)	↘NMD
Mahmud et al. (3)	20 (40)	20 (40)	14.2 ± 1.3	14.1 ± 1.5	NA	7.5 ± 1.0	7.4 ± 3.9 (F)	1	2	2	0	MICRO cutaneous (VAR)	↘PORH 5 min on fingers (*tonometry*)	NA
Mahmud et al. (43)	23 (39^‡^)	23 (23^‡^)	14.6 ± 1.7	14.7 ± 1.9	5.8 ± 3.6	8.3 ± 1.5	11.1 ± 5.5 (PP)	0	2	2	1	MICRO cutaneous (VAR)	↘PORH 5 min on fingers (*tonometry*)	NA
Nascimento et al. (44)	31 (39^‡^)	58 (41^‡^)	9.1 ± 1.8	8.4 ± 1.8	NA	9.0 ± 1.6	10.4 ± 5.5 (PP)	1	0	0	0	MACRO artery (VAR)	↘FMD 4 min on forearm (*ultrasound*)	NA
Palombo et al. (45)	16 (32)	26 (42)	18.0 ± 2.0	19.0 ± 2.0	11.0 ± 5.0	7.7 ± 1.1	9.9 ± 2.5 (F)	1	1	0	1	MICRO cutaneous (VAR)	↔ PORH 5 min on non-dominant arm (*tonometry*)	NA
Pareyn et al. (46)	34 (53)	25 (52)	15.6 ± 1.3	15.2 ± 1.7	6.3 ± 2.7	8.3 ± 1.3	10.1 ± 2.9 (PP)	1	2	2	1	MICRO cutaneous (VAR)	↘PORH 5 min on non-dominant arm (*tonometry*)	NA
Peltonen et al. (47)	10 (0)	10 (0)	33.0 ± 7.0	32.0 ± 7.0	11.0 ± 6.0	7.7 ± 0.7	9.5 ± 3.1 (PP)	1	2	0	1	MICRO muscle (VAR)	↘During maximal aerobic exercise (incremental 40 W 3 min^−1^) (*NIRS*)	NA
Pena et al. (48)	52 (42^‡^)	50 (50^‡^)	14.0 ± 2.7	14.8 ± 3.3	5.5 ± 4.0	8.9	11.1 ± 11.1 ± 4.3 (NA)	1	2	2	0	MACRO artery (VAR)	↘FMD 4 min on forearm (*ultrasound*)	↘NMD
Pichler et al. (49)	39 (50^‡^)	40 (40^‡^)	12.8 ± 2.9	12.7 ± 2.9	4.29 ± 3.0	9.2 ± 1.8	12.6 ± 4.6 (NA)	1	2	2	1	MICRO muscle (PEAK)	↘intermittent local exercise (60/min for 1 min) recovery (*NIRS associated with venous occlusion*)	NA
Pillay et al. (50)	38 (58^‡^)	28 (54^‡^)	13.0 ± 2.9	13.9 ± 2.7	5.4 ± 4.6	8.8	10.6 10.6 (NA)	0	2	2	0	MACRO artery (VAR)	↘FMD 4 min on forearm (*ultrasound*)	NA
Rissanen et al. (51)	7 (0)	10 (0)	34.8 ± 6.0	34.0 ± 7.0	15.0 ± 9.0	7.4 ± 0.9	NA (PP)	1	2	1	1	MICRO muscle (VAR)	↘maximal aerobic (incremental 40 W per 3 min) during (*NIRS*)	NA
Rodriguez-Manas et al. (52) (2 cohorts)	12 (25^‡^)	14 (50^‡^)	28.5 ± 5.9	28.4 ± 3.4	2.5 ± 3.8	6.6 ± 0.8	6.2 ± 3.5 (NA)	1	1	0	1	↘MICRO cutaneous + MICRO cutaneous + muscle (PEAK)	↔ MCh (*plethysmography*)	↔ SNP
	12 (42)	14 (50)	27.7 ± 7.6	28.4 ± 3.4	2.8 ± 3.46	11.0 ± 2.3	10.0 ± 5.2 (NA)	1	1	0	1	MICRO cutaneous + muscle (PEAK)	↘MCh (*plethysmography*)	↘SNP
Schlager et al. (53)	58 (53)	58 (41)	14.1 ± 1.7	13.6 ± 2.0	7.8 ± 3.3	7.9 ± 1.0	8.5 ± 4.7 (NA)	1	2	2	0	MICRO cutaneous (PEAK)	↗PORH 3 min (*laser Doppler*)	NA
Singh et al. (54)	31 (42)	35 (51)	15.0 ± 2.4	15.7 ± 2.7	6.8 ± 3.9	8.6 ± 1.5	8.8 ± 4.5 (NA)	1	1	1	0	MACRO artery (VAR)	↘FMD 5 min on forearm (*ultrasound*)	↔ NMD
Sochett et al. (55)	51 (51^‡^)	59 (56^‡^)	14.8	13.9	6.7	9.0 ± 1.0	9.9 ± 4.5 (NA)	1	2	2	0	MACRO artery (VAR)	↔ FMD 5 min on forearm (*ultrasound*)	NA
Tacito et al. (56)	32 (63)	28 (71)	17.3 ± 4.4	20.1 ± 5.6	4.1 ± 2.0	9.95 ± 3.0	NA (NA)	0	0	1	1	MACRO artery (VAR)	↘FMD 5 min on forearm (*ultrasound*)	NA
Tagougui et al. (57) (2 cohorts)	11^*^ (0)	11^*^ (0)	27.1 ± 6.1	25.9 ± 5.6	4.5 ± 3.6	6.6 ± 0.7	NA (PP)	1	2	2	2	MICRO muscle (VAR)	↔ During maximal aerobic exercise (incremental 20 W per 2 min) (*NIRS*)	NA
	12^*^ (42)	12^*^ (42)	25.5 ± 7.3	26.2 ± 5.0	10.9 ± 3.4	9.1 ± 0.7	NA (PP)	1	2	2	2	MICRO muscle (VAR)	↘During maximal aerobic exercise (incremental 20 W per 2 min) (*NIRS*)	NA
Tibiriça et al. (58)	48^*^ (58)	34^*^ (53)	NA	NA	NA	9.7 ± 2.5	10.5 ± 5.7 (PP)	1	2	2	2	MICRO cutaneous (PEAK)	↘PORH 3 min on forearm (*capillaroscopy*)	NA
													↘PORH 3 min on calf (*capillaroscopy*)	
Vervoort et al. (59)	39^†^ (56)	46^†^ (48)	28.1 ± 7.5	28.2 ± 6.1	8.7 ± 3.7	8.2 ± 1.2	NA (PP)	1	2	0	1	MICRO cutaneous + muscle (PEAK)	↘ACh (*plethysmography*)	NA
												MICRO muscle (PEAK)	↘during intermittent local exercise (20–30 contractions during the last min of ischemia) + PORH (*plethysmography*)	
Oral contraceptive														
Waclawovsky et al. (60)	14 (0)	5 (0)	30.3 ± 6.0	26.8 ± 5.1	NA	7.7 ± 0.75	9.3 ± 4.8 (PP)	1	2	2	0	MICRO muscle (VAR)	↔ submaximal aerobic exercise recovery (60% VO_2peak_, 40 min) (*plethysmography*)	NA
													↔ 1 RM exercise recovery (2 sec on concentric phase and 2 sec on excentric phase, 40 min) (*plethysmography*)	
Waring et al. (61)	8 (0)	8 (0)	30.0 ± 5.7	30.0 ± 5.7	NA	NA	8.5 ± 3.1 (NA)	0	2	1	1	MICRO cutaneous + muscle (PEAK)	↘Ach (*plethysmography*)	↔ SNP
Wiltshire et al. (62)	35 (49)	20 (50)	13.7 ± 2.2	13.8 ± 2.5	5.7 ± 3.3	9.1 ± 0.9	NA (NA)	1	2	2	0	MACRO artery (VAR)	↘FMD 4.5 min on forearm (*ultrasound*)	↔ NMD
Wotherspoon et al. (63)	15^*^ (27)	15^*^ (40)	39.7 ± 10.1	35.8 ± 9.7	20.6 ± 11.8	7.9 ± 0.8	12.1 ± 6.4 (NA)	1	1	0	1	MICRO cutaneous + muscle (PEAK)	↔ ACh (*plethysmography*)	↔ SNP
Yazici et al. (64)	30 (60)	29 (55)	29.0 ± 6.0	30.0 ± 6.0	7.79 ± 5.79	7.7 ± 1.31	8.0 ± 3.1 (F)	1	2	2	0	MACRO artery (VAR)	↘FMD 5 min on forearm (*ultrasound*)	↔ NMD

*Mean ± SD; HC, healthy controls; ↘the outcome was significantly lower in the group with type 1 diabetes vs. HC group; ↔ the outcome was not significantly (P > 0.05) different between the two groups; ↑ the outcome was significantly higher in the group with type 1 diabetes vs. HC group*.

*In italics: the measurement' technique*.

*Quality scores: complications 1: the absence of complication was validated (no retinopathy, no nephropathy, no neuropathy), 0: one of the three complications was not noted in the article and the authors did not respond to further inquiry. Matching 2: noticed in article, 1: visibly correct, 0: not noted in the article*.

*MICRO, means the study focused on the microcirculation; MACRO, means the study focused on macrocirculation*.

*PEAK, Data displayed as peak values in the original paper; VAR, Data displayed as variations from baseline in the original paper*.

*Occlusion-reperfusion stimuli FMD, Flow-Mediated Dilation; PORH, Post-Occlusive Reactive Hyperaemia; Pharmacological stimuli NMD, Nitroglycerin-Mediated Dilation; ACh, Acetylcholine; MCh, Methacholine*.

* means that smokers were included;

† *means that no information on smoking was provided in the article*.

‡ *means that none of the included women was taking oral contraceptives*.

*F, vascular measurements were performed during fasting; PP, vascular measurements were conducted at post-prandial state*.

*Overall, the 58 studies on endothelial function involved 15–377 participants, with a mean age ranging from 8.0 to 52.7 years, mean BMI from 18.4 to 26.6 kg.m^−2^, mean SBP from 93.9 to 126.5 mmHg, mean DBP from 57.4 to 82.5 mmHg, and mean cholesterol, HDL-cholesterol and triglycerides from 3.7 to 5.3 mmol.L^−1^, from 1.1 to 1.8 mmol.L^−1^ and from 0.7 to 3.3 mmol.L^−1^, respectively. Among participants with type 1 diabetes, mean HbA_1c_ and mean diabetes duration ranged from 6.6 to 11.9% (from 7.0 to 14.9 mmol.L^−1^) and from 2.5 to 21.1 years, respectively*.

Apparent inconsistencies about VSM and endothelial dysfunction in the literature may be related to the heterogeneity in conditions (e.g., age, glycemic control, and presence of risk factors) and vascular beds (artery, subcutaneous or muscular capillaries, and arterioles) studied as well as the different measurement methods used [ultrasonography, plethysmography, near-infrared spectroscopy (NIRS), tonometry, laser Doppler, capillaroscopy] and test stimuli applied (pharmacological substances, post-occlusive reactive hyperemia, heat). Although increased metabolic demand following physical exercise is one of the strongest physiological signals for upstream vasodilation, this natural stimulus is rarely used.

Two previous meta-analyses undertook to assess endothelial dysfunction in patients with type 1 diabetes but only in response to one type of stimulus, i.e., the response to occlusion-reperfusion at the macrovascular level [FMD (Flow mediated dilation): post-occlusive hyperemia of the brachial artery] (67) or dermal microvascular response to local thermal hyperemia (68). The latter stimuli are however only two of the numerous stimuli investigated in literature for vasoreactivity assessment. In addition, these previous meta-analyses did not take into account the presence or absence of overt vascular complications among patients included. However, in a preventive context, it appears worth dealing with patients still free from clinical complications, when one knows the strong predictive nature of endothelial dysfunction for future cardiovascular disease (5). The present meta-analysis of published data on vasodilatory capacities in patients with type 1 diabetes without complications and healthy controls was conducted to (i) evaluate the extent of early endothelial dysfunction, and assess possible concomitant VSM dysfunction, in type 1 diabetes and (ii) to disentangle, through metaregressions (i.e., sensitivity analyses), which of the many factors contributes most to the development and/or manifestation of the resulting vascular dysfunction.

## Materials and Methods

This meta-analysis was conducted according to the PRISMA Statement guidelines (69) and registered in Prospero (ID–CRD42019116319). We have followed PICOS recommendations as described throughout this section of manuscript.

### Data Sources and Searches

The searches have been undertaken using three different databases: Pubmed, Cochrane Library, and Web of Sciences until October 2019. All of the following terms, alone and in combination, were used: “type 1 diabetes,” “IDDM,” “macrovascular,” “microvascular,” “endothelial function,” “endothelium,” “exercise,” “physical activity,” “sport^*^,” “contraction,” “hemodynamic^*^,” “acetylcholine,” “sodium nitropusside,” “flow-mediated dilation,” “hyperaemia,” “iontophoresis,” “blood flow,” “blood pressure,” “FMD,” “NMD,” “vasodilation,” “vasodilatation,” “vascular,” “vasoreactivity.” We excluded the terms “type 2 diabetes,” “mice,” “mouse,” “rat.” Only articles written in English were included. Articles were selected in the first instance by title and abstract. In the second instance, a selection was performed thanks to the eligibility criteria, as described below. In the case of unclear or missing information, the authors were contacted for clarification.

### Study Selection

The main inclusion criteria were having assessed the peripheral vasodilatory capacity (dependent or not on endothelial function, i.e., corresponding to either endothelium or VSM function) in humans (men and/or women, no age limit) with type 1 diabetes free from micro- and/or macro-vascular complications (“P” from the PICOS) compared with healthy controls (“C” from the PICOS; i.e., case control studies, “S” from the PICOS). The absence of micro (retinopathy, nephropathy—i.e., albuminuria >40 mg.dL^−1^, neuropathy) and macro- (cardiac, peripheral, and cerebral) vessels complications in groups of patients with type 1 diabetes was checked based on the B category of the DCCT (exclusion of the C category).

Studies dealing with vasoreactivity in brain, heart, or retina were excluded because cerebral and cardiac vessels constitute a specific system and, contrary to peripheral vessels, the retinal vasculature is highly differentiated. Articles using needle injury methods were excluded because of additional contributions of the nervous system (21, 40). Only the studies using the following stimuli for vascular function assessment were selected: post-occlusion reactive hyperemia, local heat, physical exercise, and pharmacological substances (“I” from the PICOS). Sometimes, two stimuli were applied at the same time (12, 28, 59). In cases of interventional studies testing supplementations (32, 40, 61), drugs (61), or in a cohort study (19) only baseline values, i.e., before intervention, were analyzed in the meta-analyses.

Where the same data appeared in several publications by the same (or part of the same) group of subjects (58, 59, 63, 70–72), we opted to include the primary or the most exhaustive article (Table 1). Study selections were undertaken independently by two investigators (EL and EH). In the case of disagreements on eligibility criteria, the discrepancies were resolved by consensus with a third reviewer (PF).

### Data Extraction

The absence of vascular complications in clinical stage was carefully checked. We assessed the quality of populations matching by age, gender, and BMI (Table 1). When the authors provided only the standard error (SE) and did not respond to our request to provide standard deviations (SD), we calculated the corresponding SD, assuming the data was normally distributed.

Vascular outcomes reported corresponded either to the peak response or to a variation from baseline to peak (“O” from the PICOS). Where only peak values were provided (*cf*. in 18 studies, Table 1), we contacted the authors to calculate the corresponding variation. These data obtained from eight studies (26, 28, 46, 47, 49, 51, 59, 60) were then used in an additional analysis where only variations (either initially found in the papers or *a posteriori* calculated) were tested.

### Data Analysis

For statistical analyses we used the standardized mean difference (SMD) (Cohen's d) of endothelial or non-endothelial-dependent vascular function between the type 1 diabetes and the healthy control group. SMD allowed to standardize micro- and macro-circulation assessments as well as the large panel of measurement techniques used. Negative SMD corresponded to impaired vascular function.

We calculated weighted pooled summary estimates of SMD. For each meta-analysis, we used the DerSimonian and Laird method. Accordingly, studies were considered to be a random sample from a population of studies. Heterogeneity was assessed using *I*^2^ and chi-square heterogeneity statistics. A random-effects model was used to combine data. The overall effect was estimated using a weighted average of individual effects, with weights inversely proportional to variance in observed effects. Heterogeneity between studies was quantified using *I*^2^ statistics, with upper limits of 25, 50, and 75% as low, moderate, and high, respectively (73). Publication bias was evaluated with funnel plot and Egger's test. The pooled SMD were estimated with 95% confidence interval (CI). All analyses were performed using R software with the survival and metafor packages.

### Main Meta-Analyses (Primary Objective)

First, two meta-analyses, for endothelial function and vascular smooth muscle function, were conducted including all studies, regardless of the stimulus used or the vascular bed explored. As some studies explored the effects of more than one stimulus successively, or used more than one pharmacological substance, and some studies assessed several periods surrounding exercise (Table 1), a priority order was established (Supplementary Table S1). Overall, the stimuli inducing vasodilatation were ordered from the most physiological to the least physiological (exercise, occlusion-reperfusion, heat, and then pharmacological substances).

Exercise stimuli were either aerobic (varying from 20 to 40 min and from 45% VO_2peak_ to 100% VO_2max_) or local intermittent concentric/eccentric handgrip (duration from 30 s to 3 min; Table 1). Only one paper used two types of exercise, i.e., a session of resistance exercise (40 min) and aerobic exercise (40 min) (60).

### Metaregression Analyses (Sensitivity Analyses)

In order to disentangle, which of the many factors contributes most to the development and/or manifestation of the vascular dysfunction possibly observed in main meta-analyses, we performed metaregression analyses.

The influence of qualitative outcomes [i.e., study design (vessels size, vascular region, stimuli, technique, and types of exercise) and some subjects' characteristics (gender, generation), see Supplementary Table S2 for SMD of endothelial function and Supplementary Table S4 for SMD of VSM function] on vascular functions SMD were analyzed by comparing subgroups of studies. When a study could be included in two subgroups of the metaregression (e.g., a study analyzing separately both exercise and occlusion reperfusion stimuli in the same population), it was inserted only into the subgroup with the higher order of priority.

The influence of quantitative outcomes [i.e., the other studies' characteristics (sample size) and participant characteristics (mean age, BMI among subjects' with type 1 diabetes), and presence of concomitant risk factors (mean HbA_1c_ and duration of disease in patients; difference between patients and healthy controls in mean BMI, SBP, DBP, total cholesterol, HDL-cholesterol, and triglycerides; SMD in patients vs. controls of blood flow and diameter baseline values)] were tested using univariate metaregression analyses.

## Results

### Study Selection and Characteristics

**Figure 1:**
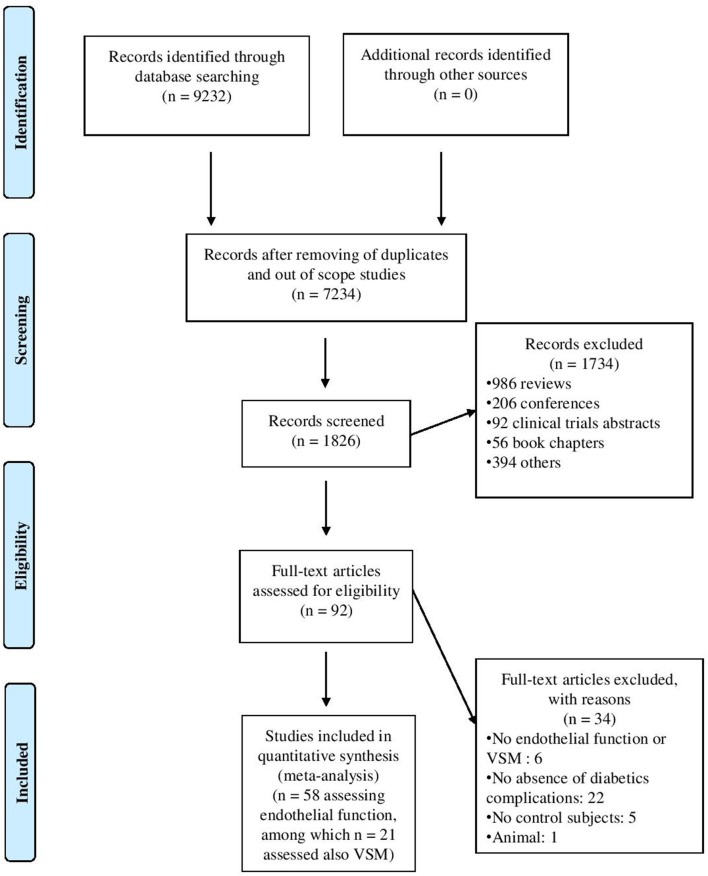
Flow-chart outlining the process of study selection.

The flow diagram (Figure 1) describes the criteria followed in order to select articles for inclusion in this meta-analysis, and 9,232 studies were identified in the first stage. After removing duplicates and out-of-scope studies, 1,826 studies were screened of which 92 articles met the inclusion criteria. Thirty-four studies were excluded after screening the abstract or reading the entire methods section. Ultimately, 58 studies among which 3 involved two (52, 57) or three (31) cohorts of patients vs. controls (in 2,322 subjects with type 1 diabetes and 1,777 healthy controls) assessing the endothelial function and 21 studies among which 1 involved 2 cohorts (52) (in 916 subjects with type 1 diabetes and 553 healthy controls) focusing on the VSM were included in the meta-analysis. The studies which measured VSM also assessed endothelial function. The studies main characteristics are reported in Table 1.

### Primary Outcomes (Main Meta-Analyses)

**Figure 2:**
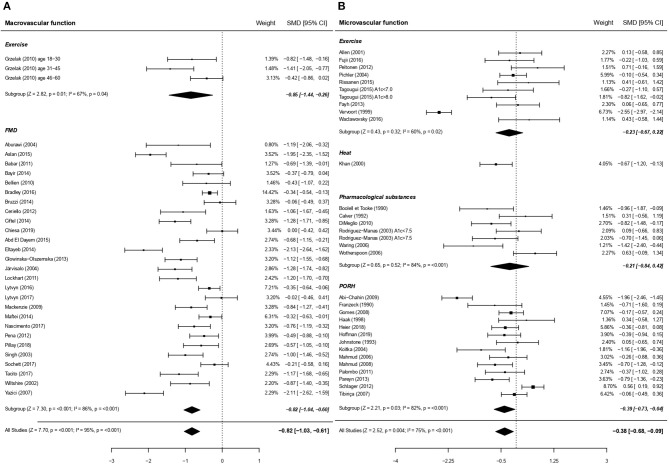
Forest plots of studies on endothelial function, according to size of vessels studied, in subjects with type 1 diabetes vs. controls. **(A)** Forest plot of studies on endothelial function focusing on macrovascular function. **(B)** Forest plot of studies on endothelial function focusing on microvascular function. In these figures the priority was set on macrocirculation over microcirculation. Similar results are obtained when priority is placed on microcirculation (SMD −0.59; 95% CI −0.74, −0.43, *P* < 0.001). PORH, Post-occlusive reactive hyperaemia; FMD, Flow Mediated Dilation. For each subgroup, the following values are indicated: *Z*- and *p*-value; *I*^2^, heterogeneity and *p* for heterogeneity. When comparing macro and microcirculation with metaregressions (for qualitative data), the difference was significant (moderator *P* = 0.001) with a greater dysfunction due to type 1 diabetes for macrovessels.

The endothelial function meta-analysis (micro- and macro-circulation pooled) revealed a significant impairment in patients with type 1 diabetes compared to healthy controls (SMD = −0.61; 95% CI = −0.79, −0.44, 4,099 subjects, *P* < 0.001), albeit with a great heterogeneity (*I*^2^ = 85.7%, *P* < 0.001; forest plot in Figure 2 and funnel plot in Supplementary Figure S1). Comparable results was obtained when only endothelial function calculated as a variation from baseline were included (*cf* data displayed in the original papers for 43 studies and *a posteriori* calculated variations for 3 additional studies) (SMD = −0.69; 95% CI = −0.88, −0.50; *P* < 0.001).

The non-endothelial (i.e., VSM) function meta-analysis demonstrated a significant impairment in type 1 diabetes compared to the control group (1,469 subjects, *P* < 0.05; forest plot in **Figure 6** and funnel plot in Supplementary Figure S2), with significant heterogeneity (*I*^2^ = 78.6%, *P* < 0.001).

Since we found some evidence of high heterogeneity, metaregressions as well as subgroup analyses were performed to determine the sources of heterogeneity. The metaregressions were also intended to explore potential moderating factors of endothelial or VSM dysfunction.

### Metaregressions (Sensitivity Analyses) for Endothelial Function

The endothelial function impairment in type 1 diabetes affected both the macrocirculation and the microcirculation (28 and 30 studies with 2,929 and 1,343 subjects, respectively; Figure 2). Macrovessels appeared to be the most affected when considering the entire population (moderator *P* = 0.001, Figures 2A,B) or adults separately (nine studies, SMD = −1.06; 95% CI = −1.51, −0.60 for macrocirculation vs. 22 studies, SMD = −0.24; 95% CI = −0.51, 0.01 for microcirculation, *P* < 0.01).

**Figure 3:**
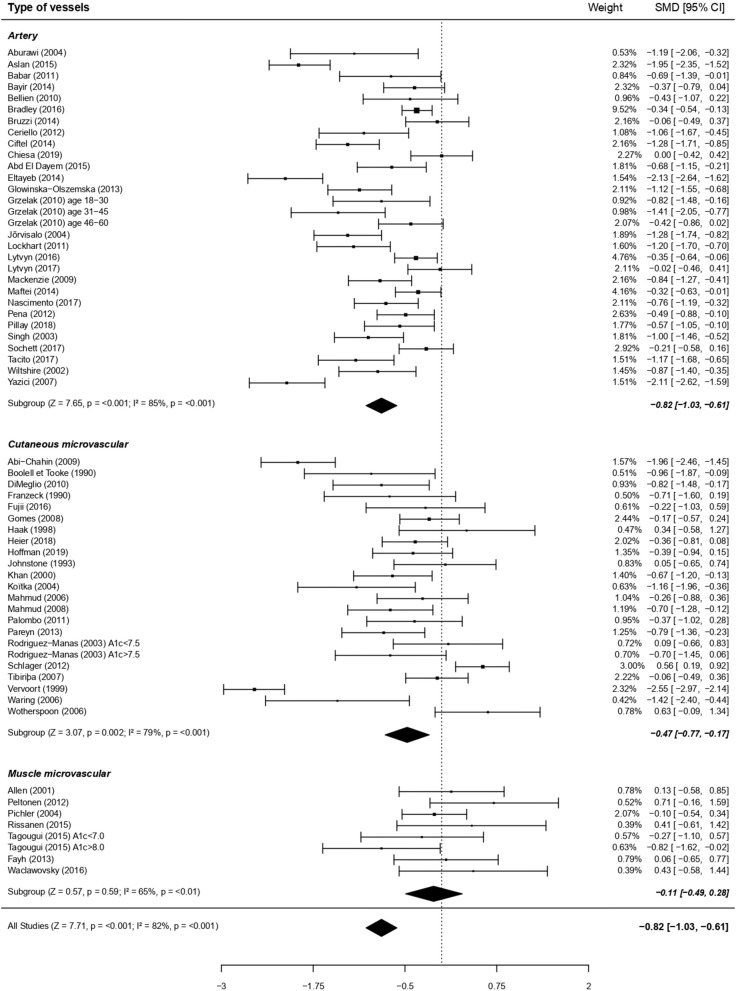
Forest plots of studies on endothelial function, according to types of vessels studied, in subjects with type 1 diabetes vs. controls. For each subgroup, the following values are indicated: *Z* and *p*-value; *I*^2^, heterogeneity and *p* for heterogeneity. When comparing the three vessels types with metaregressions (for qualitative data), the difference was significant (moderator *P* = 0.005), with a greater dysfunction for artery compared to muscle microvessels (*post-hoc* comparison using Bonferroni correction, *p* < 0.05) and a tendency for a greater dysfunction at artery compared to cutaneous microvessels (*post-hoc* comparison using Bonferroni correction, *P* = 0.09).

**Figure 4:**
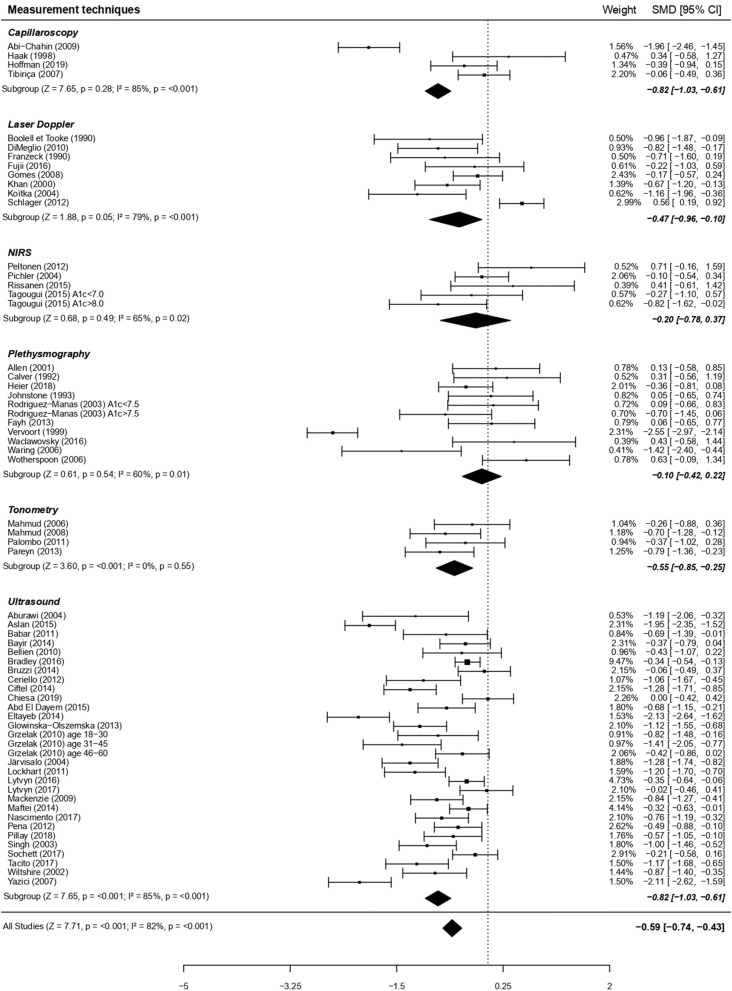
Forest plots of studies on endothelial function, according to measurement techniques, in subjects with type 1 diabetes vs. controls. For each subgroup, the following values are indicated: *Z* and *p*-value; *I*^2^, heterogeneity and *p* for heterogeneity. When comparing the six types of techniques with metaregressions (for qualitative data), the difference was significant (moderator *P* = 0.02), with a more visible dysfunction when using ultrasounds vs. plethysmography (*post-hoc* comparison using Bonferroni correction, *P* < 0.01).

In line with the more marked alteration in macrovascular vs. microvascular beds, endothelial dysfunction associated with diabetes was greater in artery compared to capillaries (Figure 3). We performed additional analyses on factors which may have contributed to this pronounced difference between macro- and micro-circulation. Noteworthy, there was an overall significant moderating effect of techniques used, translating in *post-hoc* analyses into a greater impairment of type 1 diabetes endothelial function when measured by the gold standard macrovascular technique “ultrasound” (28 studies on artery) compared to plethysmography (11 studies on cutaneous vessels; Figure 4).

The stimulus used did not seem to have a significant impact on the findings. Lower basal artery diameter in type 1 diabetes compared to controls aggravated endothelial (FMD) dysfunction (β = 1.70; *P* < 0.001).

**Figure 5:**
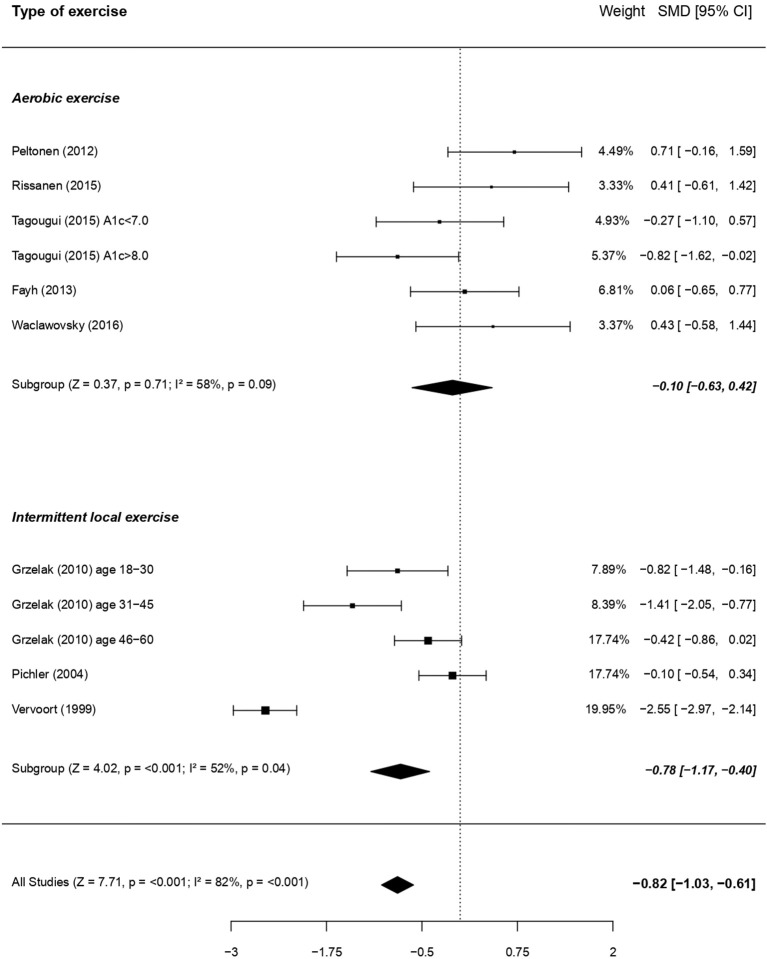
Forest plots of studies on endothelial function in response to exercise, according to the type of exercise, in subjects with type 1 diabetes vs. controls. For each subgroup, the following values are indicated: *Z* and *p*-value; *I*^2^, heterogeneity and *p* for heterogeneity. When comparing the two types of exercise with metaregressions (for qualitative data), the difference was significant (moderator *P* = 0.04), with a more visible dysfunction when using intermittent local exercise vs. aerobic exercise.

Focusing on exercise and its characteristics, impairment of vasodilatory capacity was more noticeable when regional isometric exercise (intermittent handgrip, 2 studies whose 1 including 3 cohorts) was used, compared to general aerobic exercise (constant-load for 1 study and incremental for three studies; Figure 5).

Metaregressions based on other qualitative outcomes did not show any significant results (Supplementary Tables S2, S3).

When taking into account the demographic or disease characteristics of the patients included, higher HbA_1c_ levels in patients with type 1 diabetes were associated with more pronounced endothelial dysfunction in the whole population (β = −0.20; *P* < 0.05) or when focusing only on children and adolescents (β = −0.43; *P* < 0.01).

We further examined the potential impact of concomitant risk factors differences between patients and healthy controls. Surprisingly, the difference in mean BMI between children/adolescents with type 1 diabetes and healthy controls was positively associated with endothelial function (i.e., the less BMI those in type 1 diabetes were higher, the better was the endothelial function; β = 0.39, *P* < 0.001), but with median values of mean BMI still corresponding to normal weight in these young subjects with type 1 diabetes (i.e., 20.9 kg.m^−2^). This moderating impact of BMI on endothelial dysfunction was also found in children and adolescents and in adults with type 1 diabetes when analyzing only the macrocirculation (data not shown). No other metaregression with studies' characteristics (including sample size), participant characteristics, or presence of concomitant risk factors, was significant.

### Subgroup Analyses for Endothelial Function

Considering the high heterogeneity, we have taken the care to analyze also the effect of each intervention (i.e., exercise and FMD in large vessels; exercise, heat, pharmacological substances and PORH in small vessels) separately in Figures 2A,B. For large vessels, the endothelial function, as assessed in response to exercise and FMD separately, was significantly impaired in patients with type 1 diabetes for both stimuli (*P* < 0.01 and *P* < 0.001, respectively; Figure 2A). For small vessels however, the effects of exercise and pharmacological substances separately did not reach statistical significance (*P* = 0.32, and *P* = 0.52, respectively), while endothelial dysfunction was visible in response to heat and PORH (*P* < 0.01 and *P* < 0.05, respectively).

For each stimulus in microvessels, we also analyzed separately each technique of measure (Supplementary Figure S3). For all stimuli in macrovessels (i.e., exercise and FMD) only the technique of ultrasounds was used. Overall, the heterogeneity remained high despite these subdivisions. However, focusing specifically on only one type of exercise, i.e., local intermittent exercise, allowed to reduce heterogeneity to a level close to moderate.

### Metaregressions (Sensitivity Analyses) for VSM Function

**Figure 6:**
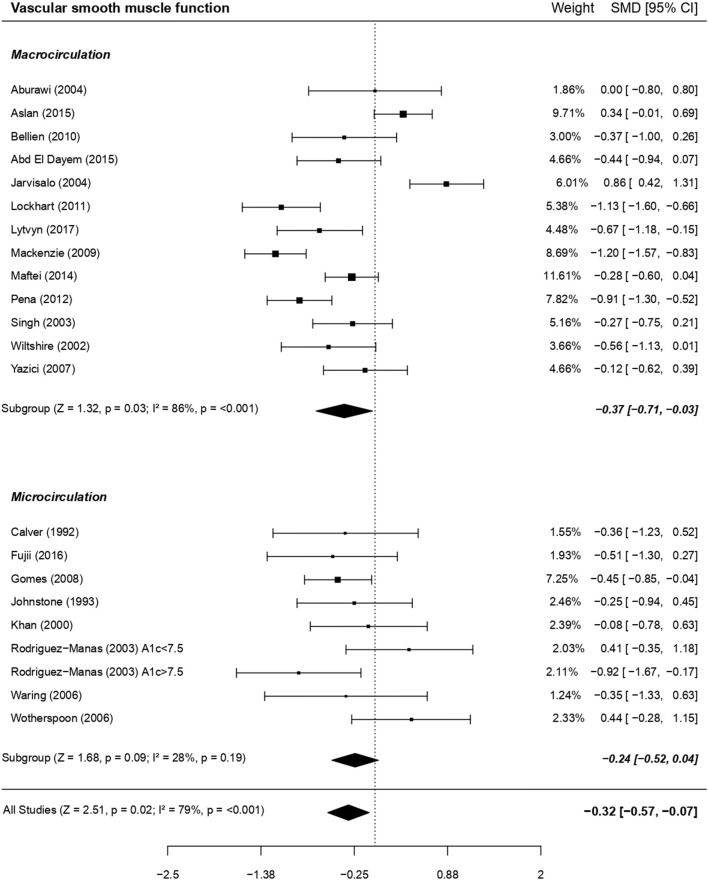
Forest plot of studies on VSM function in subjects with type 1 diabetes vs. controls. For each subgroup, the following values are indicated: *Z* and *p*-value; *I*^2^, heterogeneity and *p* for heterogeneity. There was no difference between macro and microcirculation (with metaregression for qualitative data: moderator *P* = 0.60).

Macrocirculation (i.e., brachial artery in all studies; 1,151 subjects) and microcirculation (i.e., subcutaneous capillaries in all studies; 348 subjects) VSM function was altered to the same extent in the whole population (Figure 6) as well as when focusing separately on adults or children/adolescents (data not shown). Other metaregressions based on qualitative outcomes were not significant (Supplementary Table S4).

Higher triglycerides in patients with type 1 diabetes vs. controls were determinants of a more altered VSM (metaregression between difference in mean triglycerides and VSM SMD, β = −0.80; *P* < 0.05). When focusing only on the macrovascular bed, difference in mean SBP and DBP were inversely associated with VSM SMD (β = −0.13, *P* < 0.001 and β = −0.20, *P* < 0.01, respectively). In the microvasculature, the numbers of studies focusing only on children and adolescents (*n* = 1), or taking into account HDL-C (*n* = 2) or triglycerides (*n* = 2) were too small for performing subgroup analyses using these outcomes. No other subgroup or metaregressions analyses for VSM SMD were significant.

### Comparison Between Endothelial and VSM Dysfunction

In 12 of the 21 studies where both endothelial function and VSM were measured, authors found a significant impairment only in endothelial function (Table 1). This result is in accordance with sub-analyses revealing that endothelial function (58 studies) tended to be more affected compared to VSM (21 studies; *P* = 0.08).

## Discussion

This meta-analysis, including 58 papers, attests to a medium-to-large impairment of endothelial function in patients with type 1 diabetes free from clinically relevant vascular complications. This novel result is crucial considering that ~30% of the studies on this topic failed to specifically detect this endothelial dysfunction (Table 1). Alterations of VSM function in type 1 diabetes are even less well-documented (only 8 of 21 studies attested to a significant difference; Table 1), but our current meta-analysis clearly demonstrates, for the first time, that aberrations in endothelial function is accompanied by a small-to-medium (according to Cohen's effects sizes) significant VSM dysfunction.

This impairment in VSM is alarming, considering that this dysfunction might be an even better predictor of atherosclerosis risk than endothelial dysfunction (74). Indeed, a significant negative correlation of NMD, but not FMD, with aortic intima-media thickness, a sensitive marker of atherosclerosis, has been reported in 406 adolescents with type 1 diabetes (42). In line with this notion, we found that a poorer VSM function was associated with higher triglycerides and, when focusing on macrovessels, with higher SBP and DBP. The link between VSM and these classical cardiovascular risk factors has been previously reported in type 2 diabetes (75), and may thus represent a common feature of vascular dysfunction in metabolic disease. In contrast to type 2 diabetes (75), low HDL-C did not appear to worsen VSM dysfunction in the current study. This is in line with the observation that HDL-C levels are elevated rather than decreased in type 1 diabetes (76), albeit with an alteration of their composition and function (77). Moreover, contrary to TG, HDL-C levels were described as poor predictors of micro- and macro-vascular complications in type 1 diabetes (78).

In addition to the smooth muscle dysfunction unmasked in the present meta-analysis, endothelial function tended to be still more markedly impaired. The latter result needs nevertheless cautious consideration as its assessment includes both the capacity of the endothelium to release vasodilator substances and the reactivity of the vascular smooth muscle. By the way, Khan et al. (36) revealed a correlation between iontophoresis Ach- and SNP-induced vasodilatation among children and adolescents with uncomplicated type 1 diabetes (36). Endothelial dysfunction was detected in both micro- and macro-vascular beds, albeit to a greater degree in the latter. Impaired endothelial dysfunction in macro- vs. micro-circulation in type 1 diabetes has to be considered in the context of the relative contribution of NO to the overall endothelium-dependent vasodilatation, which varies with vessels size. While NO appears crucial for vasodilatation in relatively large arteries and arterioles (79), endothelial derived hyperpolarizing factor (EDHF) makes a larger contribution to endothelial function in resistance arteries (i.e., the microcirculation) (80). Conceivably, EDHF may compensate deficiencies in NO bioactivity, as indicated in a rat model of type 1 diabetes (81). Likewise, as demonstrated in the forearm microcirculation of humans with type 1 diabetes using a cyclo-oxygenase inhibitor, prostanoid-mediated vasodilatation may compensate for a lack of NO (82). Finally, we cannot exclude that additional alterations occur in muscular vasoconstrictive capacity within arterioles, as hypothesized in a study in children and adolescents with type 1 diabetes where capillary peak perfusion during reactive hyperemia was even increased in patients vs. controls (53). In any case, our novel finding that subtle endothelial dysfunction affects more large than small vessels was unexpected, reinvigorating the debate as to whether or not microvascular complications precede macrovascular complications (83).

From a clinical perspective, it is important to note that HbA_1c_, the traditional diabetes monitoring tool, was the only one risk factor holding a significant deleterious impact on endothelial function, especially in young patients. Future studies will nevertheless be needed to confirm the conditions of this impact since we were able to evidence it only when all studies were included, but not in the adult subgroup or separately in micro and macro-vessels. Earlier studies already demonstrated a negative correlation between high HbA_1c_ and acetylcholine-induced vasodilation in children, adolescents and young adults with type 1 diabetes (36, 84) or heat-induced microvascular dilation in adolescents and young adults with type 1 diabetes (85). In one of our previous studies, we split the group with type 1 diabetes into two subgroups, with either adequate (<7%) or poor (>8%) glycemic control and demonstrated an altered exercise-induced microvascular reactivity only in the poorly-controlled group (57). Likewise, Hoffman et al. (86) revealed a more impaired reactive hyperaemic response in the human forearm of adolescents with HbA_1c_ > 8.3%.

The close link between HbA_1c_ levels and endothelial function may originate from one of two processes (i) the indirect deleterious impact of chronic hyperglycemia (as reflected by high HbA_1c_ levels) on NO bioavailability through reactive oxygen species overproduction, which inactivate NO by conversion into peroxynitrite and promote ADMA production and arginase overexpression, thus inhibiting eNOS activity and reducing the availability of the substrate L-arginine (87), (ii) a possible direct impact of glycated hemoglobin, whose affinity for NO is greater than that of the non-glycated molecule (88). This implies that, among patients with high HbA_1c_ levels, NO transported (bound on Hb) from regions of high production (i.e., the conduit arteries) may be less readily released downstream in the microcirculation, potentially altering microvascular tone.

Noteworthy, we did not detect any moderating effect of diabetes duration on endothelial dysfunction and we found that the adverse impact of HbA_1c_ was evident already in childhood and adolescence, which highlights that optimization of glycemic control should be at the center of care from the earliest stages of life onward.

Our meta-analysis did not reveal any negative influence of lipid profile or BMI on the association between endothelial dysfunction and type 1 diabetes. This result is not too surprising considering that most study participants displayed mean lipid profiles and BMI within ranges of normality (except for 1 of the 35 studies on HDL-C, 1 among the 47 studies on cholesterol). Accordingly, the majority of studies that tested the association between lipid profile and endothelial function in uncomplicated type 1 diabetes did not reveal any link between these outcomes (14, 16, 19, 45, 54, 56).

Considering the high heterogeneity in the results, we have also separated the analyses of endothelial function according to the size of vessels studied and the type of stimulus/intervention used. Separately, the effects of some stimuli (i.e., exercise and pharmacological substances) did not reach statistical significance only for small vessels. For large vessels, FMD and exercise stimuli were both associated with a significant endothelial dysfunction among the patients. In addition, when comparing the effects of the stimuli between each other, in macro as well as in microvessels, no significant differences appeared. This suggests that physical exercise, a physiological stimulus, may be efficient in triggering endothelial NO release, particularly in large vessels. In a previous study, Grzelak et al. (31) demonstrated that exercise (i.e., handgrip) was even more efficient than occlusion-reperfusion maneuver for promoting a dilatation of the artery in adults with type 1 diabetes. Only 10 studies have investigated the impact of an acute exercise stimulus on vascular function in uncomplicated patients with type 1 diabetes compared to a healthy population; 3 of these focused on skin capillaries (12, 28, 59), 7 on muscle microvasculature (26, 47, 49, 51, 57, 60) and 3 on arteries (31). When analyzing more specifically the type of exercise chosen, only one work used a session of resistance exercise and compared it to aerobic exercise of the same duration, without finding any intergroup difference of post-exercise forearm blood flow, regardless of the type of exercise performed (60). Intriguingly, concentric intermittent local (handgrip) exercise [4 cohorts; (31, 49)] appeared to induce a significantly greater vascular impairment in patients with type 1 diabetes than when performing whole-body aerobic exercise [6 cohorts; (26, 47, 51, 57, 60)]. Whether this inter-exercise difference results from a release of different and exercise-specific vasoactive molecules remains to be elucidated. Of note, isometric local exercise involves a very small active muscle mass, where blood flow is unlikely to be influenced by variations in cardiac output (89). Conversely, whole body aerobic exercise elicits substantial increases in cardiac output, and this central cardiovascular response might mask inter-group differences in peripheral vascular reactivity. Noteworthy, focusing only on local intermittent exercise allowed to considerably reduce heterogeneity to a level close to the “moderate” heterogeneity category. Finally, the ability of local isometric intermittent exercise to detect endothelial dysfunction in uncomplicated patients with type 1 diabetes provides considerable clinical perspective: such an exercise, which is physiological, cheap (requiring only a handgrip) and simple to implement, would be worth adding to the routine clinical follow-ups of uncomplicated patients, in whom pharmacological or painful (e.g., 5 min occlusion) stimuli are less well-tolerated.

### Heterogeneity

In this meta-analysis, the heterogeneity was very high in practically all the analyzed topics.

The large number of studies in this meta-analysis revealed considerable variability in methodological practice which is likely to contribute to the heterogeneity in responses observed. Factors such as the inclusion criteria and precautions before the visit or the D-day, matching between patients with type 1 diabetes and healthy controls, varied widely between studies (Table 1), inviting bias for meta-analyses. Most of the studies did match their populations only for age. Focusing on the 10 studies on exercise, only 3 indicated a matching for physical activity level. Smoking, oral contraceptives or statins could have an impact on vasoreactivity, but unfortunately this is not always considered. In addition, prevailing circulating glucose and insulin concentrations, which presumably differ considerably between subjects and studies but are regrettably not always reported (of the 58 studies, only 31 displayed glycemia, 23 reported the status of insulin injection among which 17 were in fasting and 16 in a post-prandial state), are known as modulating peripheral vasodilation (90, 91). Ultimately, while the influence of long-term glycemic control (i.e., HbA_1c_) on vascular function is systematically taken into consideration, only 2 studies (14, 48), among the 58 included in the meta-analysis, explored the moderating effect of glycemic variability (i.e., oscillating glucose concentrations) or hypoglycaemia on vascular function in patients with type 1 diabetes compared to healthy controls. These 2 studies, in children with type 1 diabetes, failed to detect significant correlation between FMD and glycemic variability, as assessed using 2-week, seven-point, self-monitored blood glucose logs (14) or 48-h continuous glucose monitoring (48). However, as previous *in vitro* (92) and *in vivo* (21, 93–98), studies suggested a possible deleterious impact of glycemic variability on vascular function, further studies are needed to better explore the strength, conditions and mechanisms of this impact. An increased magnitude of glycemic variability would generate more reactive oxygen species (including nitrotyrosine) in complications-prone cells compared to stable hyperglycemia and preliminary data suggest that protective adaptations induced by constant exposure to hyperglycemia are inadequately activated with intermittent exposure, allowing for more pronounced toxicity (92). Besides, in accordance with other reports (96, 97), Pena et al. (48) showed that an index of hypoglycaemic risk (i.e., Glycemic Risk Assessment Diabetes Equation—Hypoglycemia), measured over the 48 preceding hours, was a negative predictor of FMD but not of NMD. Noteworthy, hypoglycemia may induce vascular damages in the short and long terms: the hypoglycaemia-induced acute hemodynamic changes may precipitate and aggravate a vascular event during an acute episode (99), while repetition of hypoglycemic events could trigger abnormalities of coagulation, fibrinolysis, and inflammation.

Likewise, the combination of various ages, genders, regions assessed and, mainly, methods of assessing vascular function may greatly contribute to the high heterogeneity. In that respect, we performed metaregressions and separated sub-analyses of each intervention. Although some parameters partly explained some heterogeneity (*cf*., significant moderator *P*-values), the heterogeneity remained high in all the sub-analyses, thus limiting the extrapolation of the effect size (73). This result highlights the urgent need for vascular methods standardization. Although the gold-standard ultrasonographic assessment of large vessels by FMD is the only method benefiting from standardized guidelines (100, 101), it is operator-dependant and demands considerable practice before reproducible results are obtained (102, 103). In addition, very few studies (i.e., 1 among 30 included in the meta-analysis) took into account evoked hyperaemic shear stress while analysing FMD, while this, if altered, can constitute a reduced stimulus for dilation (104). While efforts for microvascular assessment standardization are emerging (105), further work in this direction is needed. As in this current meta-analysis, a particular technique of measure is typically appointed to correspond to either macro- or micro-vascular circulation, but in real-life these cardiovascular functions are, of course, interconnected and interdependent.

### Limits and Perspectives

While this meta-analysis focused only on peripheral vessels, vascular dysfunction is not limited to this area. Vascular dysfunction may also appear in cerebral vessels in response to aerobic exercise (106), which is highly relevant given the risk for long-term diabetes-associated cognitive decline (107).

Even if endothelial and, to a lesser extent, VSM dysfunction, have been the topic of a large number of studies in uncomplicated patients with type 1 diabetes, further studies focusing on the understanding of their underlying molecular mechanisms would benefit from agreeing on the most sensitive stimulus and most appropriate vascular bed to study for relevant routine clinical patient follow-up. NO is the main mediator controlling vascular tone and any reduction in its bioavailibility (e.g., by superoxide) translates directly into endothelial dysfunction. However, even the NO receptor (soluble guanylyl cyclase; sGC) is sensitive to oxidative stress, and a redox-driven impairment of NO/sGC signaling may contribute to VSM dysfunction. Moreover, many others pathways are involved in vasodilatation and each stimulus induces vasodilatation through common but highly specific pathways [e.g., prostaglandin (108) and EDHF/EET (epoxyeicosatrienoic acids) pathway in response to *post-occlusive reactive hyperemia*, chemical factors released by contracting skeletal muscle (109)].

Up to now, very limited data is available about underlying mechanisms of vasodilation defects in uncomplicated type 1 diabetes. Of the 55 studies included in this meta-analysis, only 4 attempted to concomitantly assess some of the putative underlying mechanisms of the vasoreactivity defect. Although Fayh et al. (26) hypothesized, by measuring total NOx (the sum of nitrite and nitrate), that NO production was unaltered in their study, this result merits confirmation because the authors did not distinguish nitrite and nitrate, although only nitrite reflects acute changes in NO synthase activity in humans (110). Using pharmacological inhibitors of endogenous NO synthesis (L-NMMA) and prostaglandin-mediated vasodilatation (indomethacin) (63) or of Ca^2+^ release in cytoplasm (35), administered concomitantly with vasoactive substances [acetylcholine or exogenous NO (sodium nitroprusside), respectively], no differences were found between patients with type 1 diabetes and healthy controls suggesting normal contribution of NO and prostaglandin as well as efficient VSM calcium channels. However, Rodriguez-Manas et al. (52) suggested the involvement of oxidative stress in endothelial dysfunction among poorly-controlled type 1 diabetes subjects: they indeed demonstrated an improvement of blunted vasodilatory response to metacholine when co-infusing superoxide dismutase in patients with HbA_1c_ ≥ 7.5%, while no changes appeared for healthy controls or well-controlled patients.

In addition to help selecting the most appropriate stimulus for routine follow-ups of vascular function in type 1 diabetes, future efforts should focus on a better understanding of the underlying mechanisms to design specific interventions and molecular target to slow down progressive vascular damage. Several studies already tested some non-pharmacological interventions in patients with uncomplicated type 1 diabetes and displayed encouraging results for regular physical exercise (111) and nutritional supplementation with L-arginine (26) and vitamin C (112).

In conclusion, this meta-analysis not only corroborates the presence of a medium-to-large impairment of endothelial function, even in response to physiological stimuli such as exercise, but also highlights a VSM dysfunction in children and adults with type 1 diabetes free from clinical vascular complications. Of note, heterogeneity was high and was not further explained by subgroups analyses, thus limiting the extrapolation of the effect size and highlighting the urgent need for vascular methods standardization. Surprisingly, endothelial dysfunction seemed more marked in large rather than small blood vessels, re-invigorating the debate about the timing and complexity of the development of vascular complications in type 1 diabetes. The inverse relationship between endothelial function and HbA_1c_ provides further arguments for identifying therapeutic strategies aimed at improving glycemic control.

## Data Availability Statement

All datasets generated for this study are included in the article/Supplementary Material.

## Author Contributions

EL and EH extracted and analyzed data and wrote the manuscript. LD performed statistical analyses. PF gave his clinical expertise to the inclusion of studies in the meta-analysis and resolved discrepancies by consensus in the case of disagreements on eligibility criteria between EL and EH. PF, LD, CB, MF, JB, SB, MP-C, and MR were involved in interpreting data and reviewing the manuscript. EH is the guarantor of this work and, as such, had full access to all data and takes responsibility for the integrity of the data and the accuracy of the data analysis.

## Conflict of Interest

The authors declare that the research was conducted in the absence of any commercial or financial relationships that could be construed as a potential conflict of interest.

## Abbreviations

ach: Acetylcholine
ADMA: Asymmetrical dimethylarginine
BMI: Body Mass Index
cGMP: Cyclic guanosine monophosphate
CI: Confidence Interval
DBP: Diastolic Blood Pressure
EDHF: Endothelial Derived Hyperpolarizing Factor
EET: Epoxyeicosatrienoic acids
eNOS: Endothelial Nitric Oxide Synthase
FMD: Flow-mediated dilation
HbA1c: Glycated Hemoglobin
HC: Healthy Controls
HDL-C: High Density Lipids-Cholesterol
IDDM: Insulin-Dependent Diabetes Mellitus
L-NMMA: NG-Monomethyl-L-arginine
MACRO: Macrocirculation
MCh: Metacholine
MICRO: Microcirculation
NIRS: Near-Infrared Spectroscopy
NMD: Nitrate-Mediated Dilation
NO: Nitric Oxide
NOx: Sum of nitrite and nitrate
PEAK: Data displayed as peak values in the original paper
PORH: Post-occlusive reactive hyperaemia
PP: Post-Prandial
SBP: Systolic Blood Pressure
SD: Standard Deviation
SE: Standard Error
sGC: Guanylate cyclase
SMD: Standard Mean Deviation
SNP: Sodium Nitropusside
TG: Tryglycerides
VAR: Data displayed as variations from baseline in the original paper
VO2: Aerobic capacity
VSM: Vascular smooth muscle.
