# Supplementary figures and images for: Early Endothelial Dysfunction in Type 1 Diabetes Is Accompanied by an Impairment of Vascular Smooth Muscle Function: A Meta-Analysis
Source: Front Endocrinol (Lausanne). 2020 Apr 17;11:203. doi: 10.3389/fendo.2020.00203 (PMC7180178; doi:10.3389/fendo.2020.00203)

# Endothelium-dependent vascular function

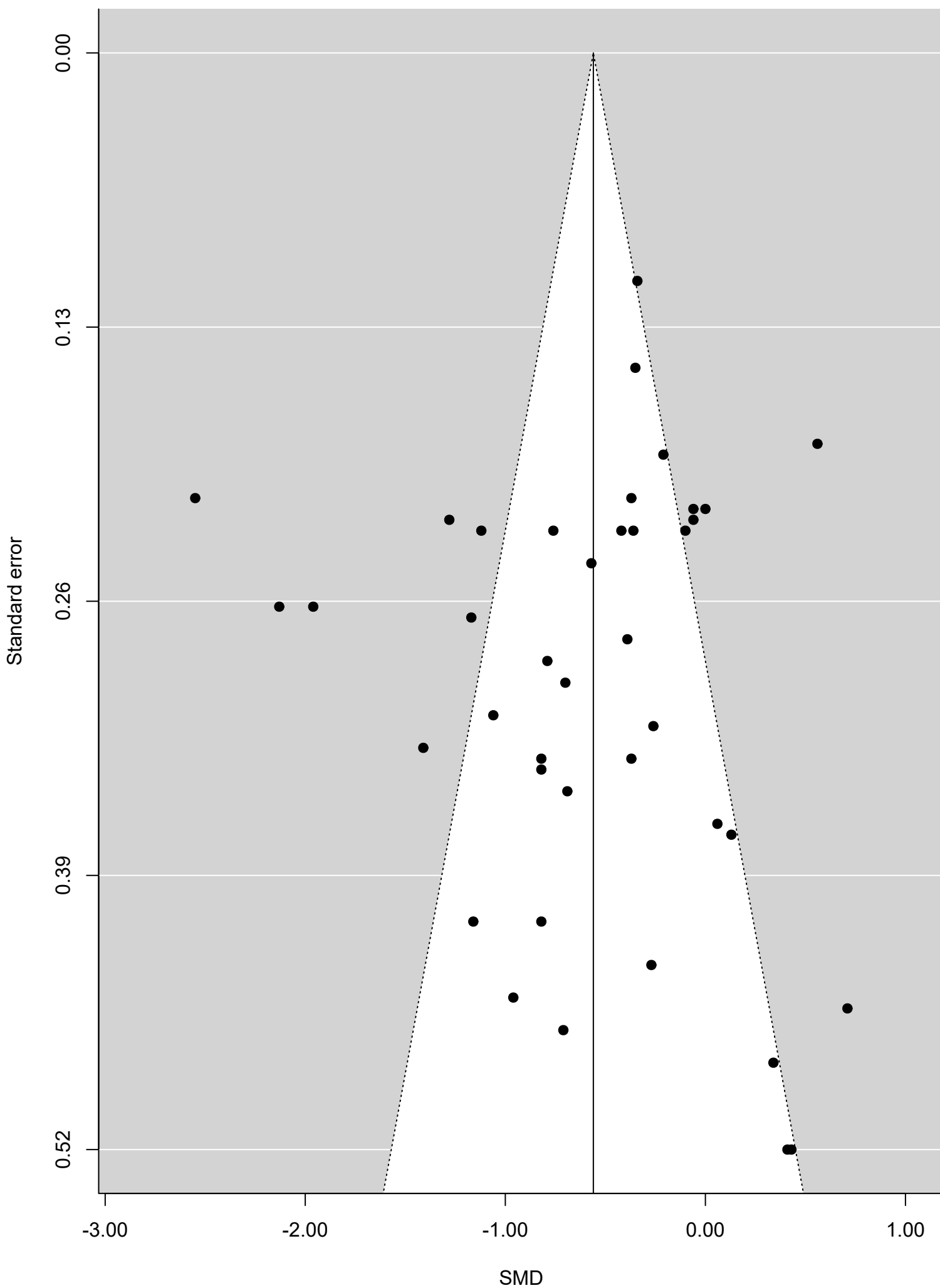

Supplement: Supplementary file 1 [file Image_1.pdf]

# Vascular smooth muscle function

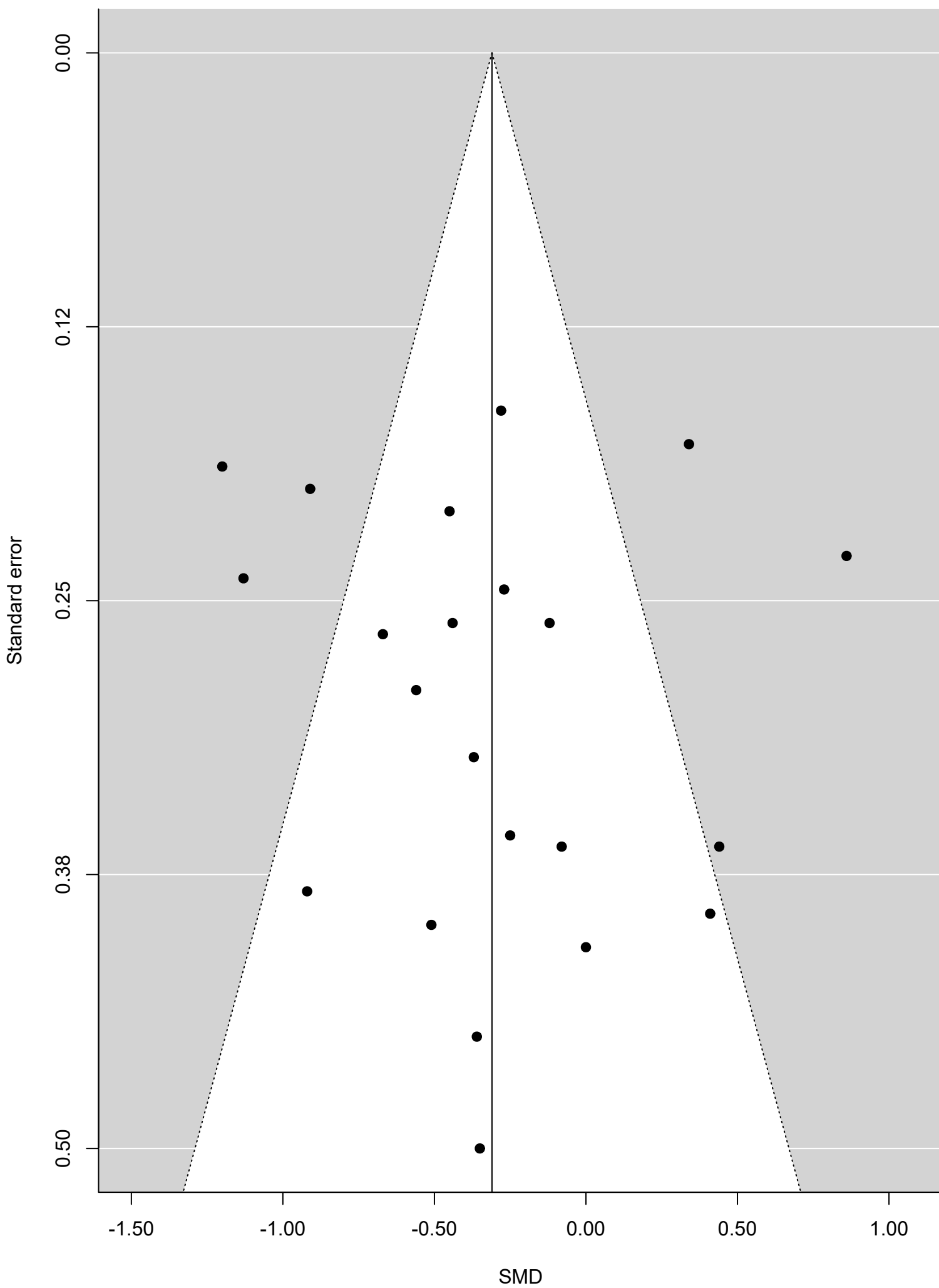

Supplement: Supplementary file 2 [file Image_2.pdf]
