# Supplementary material for: Early Endothelial Dysfunction in Type 1 Diabetes Is Accompanied by an Impairment of Vascular Smooth Muscle Function: A Meta-Analysis
Source: Front Endocrinol (Lausanne). 2020 Apr 17;11:203. doi: 10.3389/fendo.2020.00203 (PMC7180178; doi:10.3389/fendo.2020.00203)
Supplement: Supplementary file 3 [file Image_3.pdf]

S3a – Techniques of exercise in microcirculation

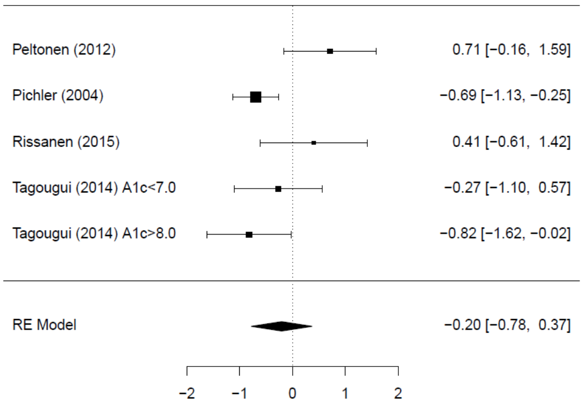

NIRS – Z = -0.69, p = 0.49, I^2 = 65%, p_{het} = 0.02

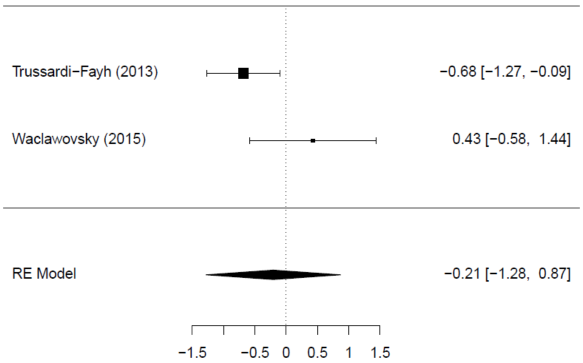

Plethysmography – Z = -0.37, p = 0.71, I^2 = 71%, p_{het} = 0.06

S3b – Techniques of PORH in microcirculation

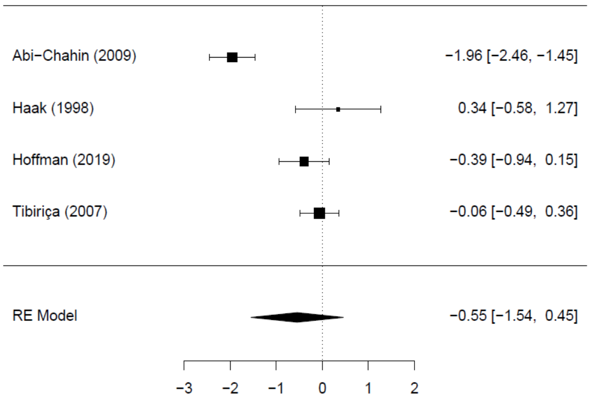

Capillaroscopy – Z = -1.07, p = 0.28, I^2 = 92%, $p_{het} < 0.001$

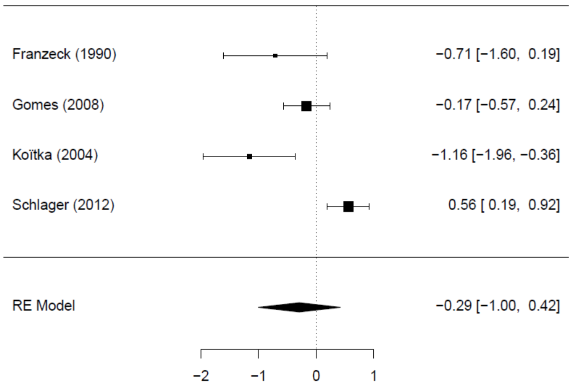

Laser Doppler – Z = -0.81, p = 0.42, I^2 = 85%, $p_{het} < 0.001$

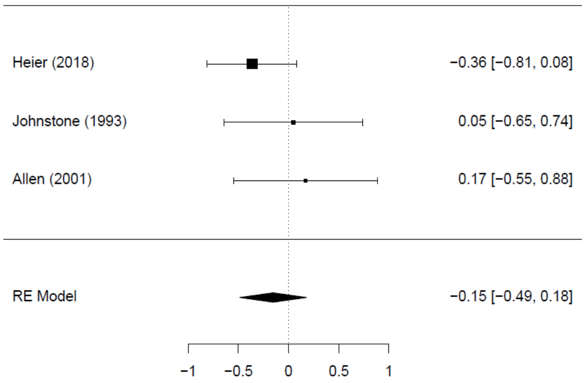

Plethysmography – Z = -0.91, p = 0.36, I^2 = 0%, p_{het} = 0.38

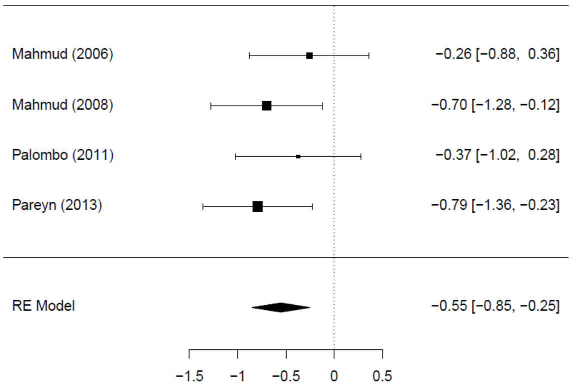

Tonometry – Z = -3.6, $p < 0.001$, I^2 = 0%, p_{het} = 0.55

S3c – Techniques of pharmacological substances in microcirculation

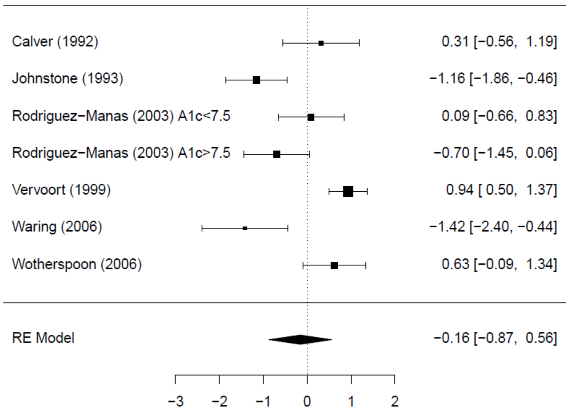

Plethysmography – Z = -0.43, p = 0.67, I^2 = 86%, $p_{het} < 0.001$

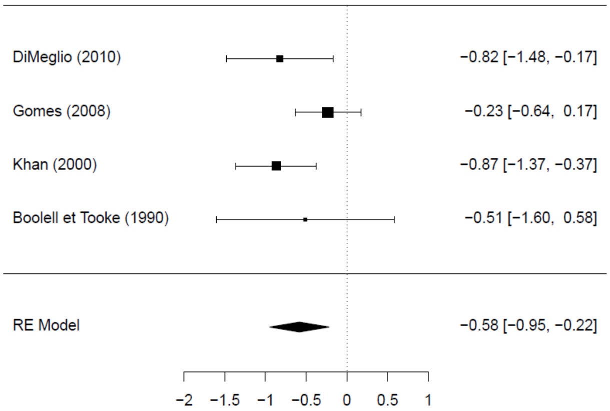

Laser Doppler – Z = -3.14, p = 0.002, I^2 = 35%, p_{het} = 0.21
