# Supplementary material for: Early Endothelial Dysfunction in Type 1 Diabetes Is Accompanied by an Impairment of Vascular Smooth Muscle Function: A Meta-Analysis
Source: Front Endocrinol (Lausanne). 2020 Apr 17;11:203. doi: 10.3389/fendo.2020.00203 (PMC7180178; doi:10.3389/fendo.2020.00203)
Supplement: Supplementary file 4 [file Table_1.DOCX]

***Supplementary data:***

**Supplementary Figure S1:** Funnel Plot of studies focusing on endothelial function

Legend. This graphic is focused on endothelial function in subjects with type 1 diabetes and apparently healthy controls (order of priority: macrocirculation)

**Supplementary Figure S2:** Funnel Plot of studies focusing on vascular smooth muscle function

Legend. This graphic is focused on VSM function in subjects with type 1 diabetes and apparently healthy controls (order of priority: macrocirculation).

**Supplementary Figure S3:** Subgroup analyses based on measurements techniques for each stimulus on microvessels

Figure S3a – Techniques of exercise in microcirculation

Figure S3b – Techniques of PORH in microcirculation

Figure S3c – Techniques of pharmacological substances in microcirculation

**Supplementary Table S1:** Details of priority order

| **Number of priority** | **Priority order of stimuli** | **Vascular region assessed** |
| --- | --- | --- |
| 1 | Maximal aerobic exercise: measurement during exercise | Muscle microvascular |
| 2 | Submaximal aerobic exercise: measurement during exercise | Muscle microvascular |
| 3 | Intermittent local exercise + post-occlusive reactive hyperemia: measurement during exercise | Cutaneous microvascular |
| 4 | Submaximal aerobic exercise + heat: measurement at the immediate end of exercise | Cutaneous microvascular |
| 5 | Submaximal aerobic exercise: measurement at the immediate end of exercise | Muscle microvascular |
| 6 | Intermittent local exercise: measurement at the immediate end of exercise | Brachial artery |
| 7 | post-occlusive reactive hyperemia: arm or hand | Cutaneous microvascular + brachial artery |
| 8 | post-occlusive reactive hyperemia: feet | Cutaneous microvascular |
| 9 | Heat | Cutaneous microvascular + brachial artery + radial artery |
| 10 | Pharmacological substance: Substance P, Metacholine, Acetylcholine, Capsaicin* | Cutaneous microvascular |
| 11 | Submaximal aerobic exercise + heat: during the recovery | Cutaneous microvascular |
| 12 | Submaximal aerobic exercise: during the recovery | Brachial artery |
| 13 | Intermittent local exercise: during the recovery | Muscle microvascular + brachial artery |

Legend: * One study (1) investigated the effects of two pharmacological sustances, *i.e.* substance P and capsaicin, in the same population. We opted to prioritise substance P over capsaicin since the former is known to act directly on the NO pathway (1) while the latter acts indirectly via TRPV1-activated production of prostaglandins (2). While we chose to set the priority order based on the more physiological stimulus to the less one, other order of priorities might also be defensible. Another possibility would be to rank the stimuli according to their putative ability to discriminate endothelial vs. non endothelial components (e.g. heat, pharmacological substances, occlusion-reperfusion, and exercise). Using the latter, we found reasonably comparable results for total analyses, sub-analyses and meta-regressions compared to those presented in the current manuscript.

**Supplementary table S2:** Subgroup analyses on SMD of endothelial function in type 1 diabetes subjects compared to heathy controls.

| **Subgroups for EF SMD** | **Number of studies** | **SMD (95% CI)** | ***I²*_heterogeneity_** | ***P*_heterogeneity_** | ***P*_moderator_** | **References** |
| --- | --- | --- | --- | --- | --- | --- |
| **Stimuli assessed** |  |  |  |  |  |  |
| Occlusion-reperfusion | 41 | -0.67 (-0.86, -0.48) | 86 | 0.001 | 0.24 | (2, 3, 17, 18, 20, 22, 27, 37, 47, 54-58, 62, 65, 83, 86-90, 92, 94, 95, 97-112) |
| Heat | 1 | -0.67 (-1.20, -0.13) | NA | NA |  | (42) |
| Exercise | 10 | -0.45 (-0.80, -0.09) | 74 | 0.001 |  | (14-16, 26, 28-31, 34, 35) |
| Pharmacological substances | 8 | -0.21 (-0.84, 0.42) | 84 | 0.001 |  | (19, 25, 33, 42, 91, 93, 96, 100) |
| **Focus on exercise** (excluding studies employing two simultaneous stimuli) |  |  |  |  |  |  |
| Measurement during exercise | 4 | -0.02 (-0.71, 0.67) | 60 | 0.06 | 0.15 | (28, 30, 34) |
| Measurement at the end of exercise | 2 | -0.79 (-1.21, -0.38) | 52 | 0.10 |  | (26, 35) |
| Measurement on the recovery | 2 | -0.23 (-1.31, 0.85) | 75 | 0.05 |  | (29, 31) |
| **Demographic characteristics - Gender** |  |  |  |  |  |  |
| Mixed population | 49 | -0.63 (-0.80, -0.46) | 84 | 0.001 | 0.15 | (2, 3, 15-18, 20, 22, 25, 27, 29, 33, 34, 37, 42, 47, 54-58, 62, 65, 83, 86-92, 94-112) |
| Male Gender only | 9 | -0.32 (-0.72, 0.08) | 69 | 0.001 |  | (14, 19, 26, 28, 30, 31, 34, 35, 93) |
| **Demographic characteristics -Generation** |  |  |  |  |  |  |
| Adults | 31 | -0.53 (-0.79, -0.26) | 84 | 0.001 | 0.53 | (14-19, 22, 25, 28, 30, 31, 33-35, 55, 56, 58, 65, 83, 87, 89, 91, 93, 98, 100, 101, 103, 104) |
| Adolescents & children | 28 | -0.63 (-0.82, -0.45) | 80 | 0.001 |  | (2, 3, 20, 27, 29, 37, 42, 47, 54, 57, 62, 86, 88, 90, 92, 94-97, 99, 102, 105-111) |

**Legend**

Negative SMD corresponded to impaired endothelial function in patients with type 1 diabetes compared with healthy controls.

When several measurements were performed in the same paper (15, 31), the highest priority was given to those obtained during the exercise, followed by measurements at the immediate end of exercise and then during recovery.

Pairwise post-hoc comparisons using Bonferoni corection: **P*<0.05; ***P*<0.01

*P*_moderator_ means p-value of the subgroup interaction

**Supplementary table S3:** Subgroup analysis on SMD of endothelial function in type 1 diabetes subjects compared to controls.

| **MACROCIRCULATION EF SMD Subgroup** | **Number of studies** | **SMD (95% CI)** | ***I²*_heterogeneity_** | ***P*_heterogeneity_** | ***P*_moderator_** | **References** |
| --- | --- | --- | --- | --- | --- | --- |
| **Stimuli assessed** |  |  |  |  |  |  |
| Occlusion-reperfusion | 27 | -0.82 (-1.04, -0.60) | 86 | 0.001 | 0.91 | (2, 18, 20, 35, 37, 54, 55, 57, 58, 62, 65, 86, 88-90, 92, 94, 95, 97, 99, 104-106, 108-112) |
| Exercise | 1 | -0.85 (-1.44, -0.26) | 68 | 0.04 |  | (35) |
| **Demographic characteristics - Gender** |  |  |  |  |  |  |
| Mix population | 27 | -0.82 (-1.04, -0.60) | 86 | 0.001 | 0.91 | (2, 18, 20, 37, 54, 55, 57, 58, 62, 65, 86, 88-90, 92, 94, 95, 97, 99, 104-106, 108-112) |
| Men only | 1 | -0.85 (-1.44, -0.26) | 68 | 0.04 |  | (35) |
| **Demographic characteristics - Generation** |  |  |  |  |  |  |
| Adult | 8 | -1.06 (-1.52, -0,60) | 87 | 0.001 | 0.10 | (18, 35, 55, 58, 65, 89, 104, 112) |
| Adolescents & children | 20 | -0.70 (-0.91, -0,49) | 81 | 0.001 |  | (2, 20, 37, 54, 57, 62, 86, 88, 90, 92, 94, 95, 97, 99, 105, 106, 108-111) |
| **MICROCIRCULATION EF SMD Subgroup** | **Number of studies** | **SMD (95% CI)** | ***I²*_heterogeneity_** | ***P*_heterogeneity_** | ***P*_moderator_** | **References** |
| **Type of vessels assessed** |  |  |  |  |  |  |
| Cutaneous microvascular | 18 | -0.47 (-0.77, -0.17) | 79 | 0.001 | 0.16 | (3, 14, 15, 17, 21, 27, 42, 47, 56, 83, 87, 91, 96, 98, 100-103, 107) |
| Muscle microvascular | 8 | -0.11 (-0.49, 0.28) | 65 | 0.004 |  | (14, 16, 26, 28-31, 34) |
| **Type of vascular analyses** |  |  |  |  |  |  |
| Data displayed as variations from baseline in the original paper | 14 | -0.49 (-0.82, -0.16) | 72 | 0.001 | 0.16 | (3, 15, 27, 28, 30, 31, 34, 42, 56, 87, 96, 98, 101, 107) |
| Data displayed as peak values in the original paper | 16 | -0.19 (-0.46, 0.08) | 71 | 0.001 |  | (14, 16, 17, 19, 22, 25, 26, 29, 33, 47, 83, 91, 93, 100, 103) |
| **Stimuli assessed** |  |  |  |  |  |  |
| Occlusion-reperfusion | 15 | -0.39 (-0.73, -0.04) | 82 | 0.001 | 0.84 | (3, 14, 17, 22, 27, 47, 56, 83, 87, 98, 100-103, 107) |
| Heat | 1 | -0.67 (-1.20, -0.13) | 0 | 1 |  | (42) |
| Exercise | 7 | -0.23 (-0.67, 0.22) | 60 | 0.02 |  | (26, 28-31, 34) |
| Pharmacological substances | 8 | -0.21 (-0.84, 0.42) | 84 | 0.001 |  | (16, 25, 33, 83, 91, 93, 100) |
| **Demographic characteristics - Gender** |  |  |  |  |  |  |
| Mix population | 23 | -0.41 (-0.66, -0.15) | 78 | 0.001 | 0.22 | (3, 15-17, 22, 25, 27, 29, 33, 34, 42, 47, 56, 83, 87, 91, 96, 98, 100-103, 107) |
| Men only | 7 | -0.07 (-0.53, 0.40) | 60 | 0.01 |  | (14, 19, 26, 28, 30, 34, 93) |
| **Demographic characteristics - Generation** |  |  |  |  |  |  |
| Adults | 22 | -0.30 (-0.59, -0,02) | 76 | 0.001 | 0.51 | (14-17, 19, 22, 25, 26, 28, 30, 31, 33, 34, 56, 83, 87, 91, 93, 98, 100, 101, 103) |
| Adolescents & children | 8 | -0.45 (-0.85, -0,05) | 79 | 0.001 |  | (3, 27, 29, 42, 47, 96, 102, 107) |

Legend:

This table are focused on the subgroup of macrovascular function and of the subgroup of microvascular function in endothelial function.

Negative SMD corresponded to impaired endothelial function in patients with type 1 diabetes compared with healthy controls.

All studies focusing on macrovessels reported variation values from baseline, so that no comparison with peak values could be performed.

**Supplementary table S4:** Subgroup analysis on SMD of VSM in type 1 diabetes subjects compared to heathy controls.

| **VSM SMD Subgroup** | **Number of studies** | **SMD (95% CI)** | ***I²*_heterogeneity_** | ***P*_heterogeneity_** | ***P*_moderator_** | **References** |
| --- | --- | --- | --- | --- | --- | --- |
| **Type of vascular analyses** |  |  |  |  |  |  |
| Data displayed as peak values in the original paper | 7 | -0.22 (-0.57, 0,13) | 43 | 0.11 | 0.60 | (2, 19, 25, 33, 93, 100) |
| Data displayed as variations from baseline in the original paper | 15 | -0.36 (-0.67, -0,05) | 84 | 0.001 |  | (2, 15, 18, 37, 42, 55, 57, 62, 86, 88, 89, 104, 106, 111, 112) |
| **Techniques measurements** |  |  |  |  |  |  |
| Ultrasound (macro) | 13 | -0.37 (-0.71, -0.03) | 86 | 0.001 | 0.80 | (2, 18, 37, 55, 57, 62, 86, 88, 89, 104, 106, 111, 112) |
| Plethysmography (micro) | 5 | -0.15 (-0.60, 0.28) | 45 | 0.10 |  | (19, 25, 33, 83, 93) |
| Laser Doppler (micro) | 3 | -0.38 (-0.70, -0.06) | 0 | 0.63 |  | (15, 42, 100) |
| **Demographic characteristics - Gender** |  |  |  |  |  |  |
| Mixed population | 19 | -0.32 (-0.58, -0,06) | 81 | 0.001 | 0.94 | (2, 15, 18, 25, 33, 37, 42, 55, 57, 62, 83, 86, 88, 89, 100, 104, 106, 111, 112) |
| Men only | 2 | -0.36 (-1.01, 0,30) | 0 | 0.99 |  | (19, 93) |
| **Demographic characteristics - Generation** |  |  |  |  |  |  |
| Adults | 11 | -0.30 (-0.60, -0,01) | 68 | 0.001 | 0.90 | (15, 18, 19, 25, 33, 55, 83, 89, 93, 100, 104, 112) |
| Adolescents & children | 10 | -0.33 (-0.76, 0,09) | 86 | 0.001 |  | (2, 37, 42, 57, 62, 86, 88, 106, 111) |

Legend

Negative SMD corresponded to impaired VSM function in patients with type 1 diabetes compared with healthy controls.
